# Photo‐Biomodulation of the Hippocampus Using Near‐Infrared Laser to Enhance Cognitive Function in Mice

**DOI:** 10.1002/advs.202417380

**Published:** 2025-04-07

**Authors:** Wei‐Tong Pan, Han‐Wen Gu, Yan‐Bo Zhou, Sophie Ma, Daqing Ma, Pan‐Miao Liu, Jian‐Jun Yang

**Affiliations:** ^1^ Department of Anesthesiology Pain and Perioperative Medicine The First Affiliated Hospital of Zhengzhou University Zhengzhou 450052 China; ^2^ Neuroscience Research Institute Zhengzhou University Academy of Medical Sciences Zhengzhou 450052 China; ^3^ Perioperative and Systems Medicine Laboratory and Department of Anesthesiology Children's Hospital Zhejiang University School of Medicine National Clinical Research Center for Child Health Hangzhou 310053 China; ^4^ Division of Anaesthetics Pain Medicine and Intensive Care Department of Surgery & Cancer Faculty of Medicine Imperial College London Chelsea and Westminster Hospital London SW10 9NH UK

**Keywords:** cognitive function, dentate gyrus, neuronal activity, photo‐biomodulation

## Abstract

Photo‐biomodulation (PBM) with near‐infrared (NIR) laser emerges as a promising noninvasive therapy for enhancing cognitive function. Here the effects of PBM, equipped with an 808 nm NIR laser, on cognitive function are investigated when used to irradiate hippocampi of mice. NIR irradiation once a day for 5 min per day for 5 consecutive days significantly improves the learning and memory performance of naïve mice assessed with the Barnes and Y maze tests. PBM also significantly attenuates cognitive dysfunction following anesthesia and surgery, and lipopolysaccharide challenge, respectively. Cognitive enhancements are associated with increased neuronal oscillations and enhanced theta–gamma coupling in the hippocampal dentate gyrus (DG) during cognitive processing. High neuronal activation in the DG is evidenced by increased c‐Fos expression and calcium signal intensity. The improvements are also associated with high‐density cells in the DG together with facilitating local energy deposition and neuronal activation. NIR‐laser‐activated axonogenesis and oxidative phosphorylation‐related genes assessed with single‐cell RNA sequencing were seen in the DG. These findings suggest that PBM very likely holds great potential as a noninvasive therapy for cognitive dysfunction.

## Graphical abstract

Here a transcranial photo‐biomodulation approach is developed using near‐infrared (NIR) laser on hippocampus of mice, revealing significant improvements in learning and memory ability of mice under normal and disease conditions. The improvement results from the high‐density cell‐induced NIR local energy deposition, neuronal activation, and axonogenesis in the hippocampal dentate gyrus, suggesting a universal finding for the photo‐biomodulation in brain.

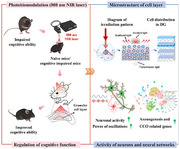

## 1 Introduction

Cognitive function is the brain's ability to judge the features, states, and interrelations of objective things. Once the brain suffers from damage or injury under pathological conditions and/or neurological diseases, cognitive dysfunction may occur and manifests with abnormal learning, memory, and thinking judgment, accompanied by aphasia, apraxia, agnosia, or dyslexia.^[^ ^1^ ^]^ Underlying mechanisms are largely unknown, although cognitive dysfunction is related to abnormalities in protein expression, synaptic connections, and neuronal activity of various brain regions.^[^ ^2^ ^]^ Although conventional treatment such as drug therapy or brain stimulation via craniotomy can achieve some effects, the unavoidable side effects of medication as well as the invasive trauma from surgery make these therapies suboptimal. Accordingly, physical intervention techniques may be good alternatives in the treatment of functional disease conditions.^[^ ^3^ ^]^ Among those, electromagnetic fields or light therapy have been used as alternative treatments clinically.^[^ ^4^ ^]^ For example, transcranial electrical stimulation and transcranial magnetic stimulation achieved certain clinical benefits in cognition‐related diseases or conditions, such as postoperative delirium and depression.^[^ ^5^ ^]^ However, the treatments are often accompanied with some inevitable complications including headaches and even epilepsy.^[^ ^6^ ^]^ Therefore, developing new intervention therapies are urgently needed.

As such, photo‐biomodulation (PBM) utilizing the photon energy of light to modulate the brain functions of animals or humans is promising.^[^ ^7^ ^]^ In this therapy, near‐infrared (NIR) laser (780–1100 nm) is used to modulate brain physiological functions. It is benefited from effective penetrability and excellent photobiological regulatory ability, generated from photon energy absorbed by cytochrome C oxidase (CCO) of mitochondria.^[^ ^8^ ^]^ Accordingly, NIR laser has been reported preclinically and clinically to improve cognition and treat depression in various disease models or neurological diseases, respectively. This is owing to its functions of reducing scavenging oxygen free radicals and anti‐inflammatory effects, reducing amyloid protein deposition and enhancing neurotrophic factors’ actions.^[^ ^8^, ^9^ ^]^ Based on previous studies, our work further explored the mechanism of NIR laser improving cognitive function in mice.

The hippocampus is one of the most closely related encephalic regions and is responsible for the spatial learning and memory components of cognitive function. Targeting the hippocampus with NIR laser showed a significant improvement in dementia patients, while tolerance and no adverse side effects were reported.^[^ ^10^ ^]^ However, the effectiveness for NIR in other cognitive dysfunction‐related symptoms, such as perioperative neurocognitive dysfunction (PND), has not been studied. PND is a common postoperative complication in elderly patients, which greatly affects the prognosis of patients.^[^ ^11^ ^]^ However, there is still a lack of effective prevention and treatment methods in clinic. Surgical anesthesia and inflammatory infection are considered to be the two main causes of PND, and both are associated with impaired hippocampal function.^[^ ^12^ ^]^ Therefore, we selected postoperative cognitive dysfunction (POCD) model and lipopolysaccharide (LPS)‐induced cognitive dysfunction model to explore the prevention and treatment effect of NIR on PND, and attempted to develop a noninvasive physical technique for perioperative brain protection.

In this study, we developed a PBM equipped with 808 nm NIR laser in order to directly and noninvasively irradiate the hippocampus region of mice during learning and memory processes. Thus, we explored its effect and therapeutic usefulness in naïve mice and the mouse models of POCD‐ as well as LPS‐induced cognitive dysfunctions and underlying mechanisms.

## 2 Results

### 2.1 808 nm NIR Laser Improves the Cognitive Function of the Naïve Mice

**Figure 1:**
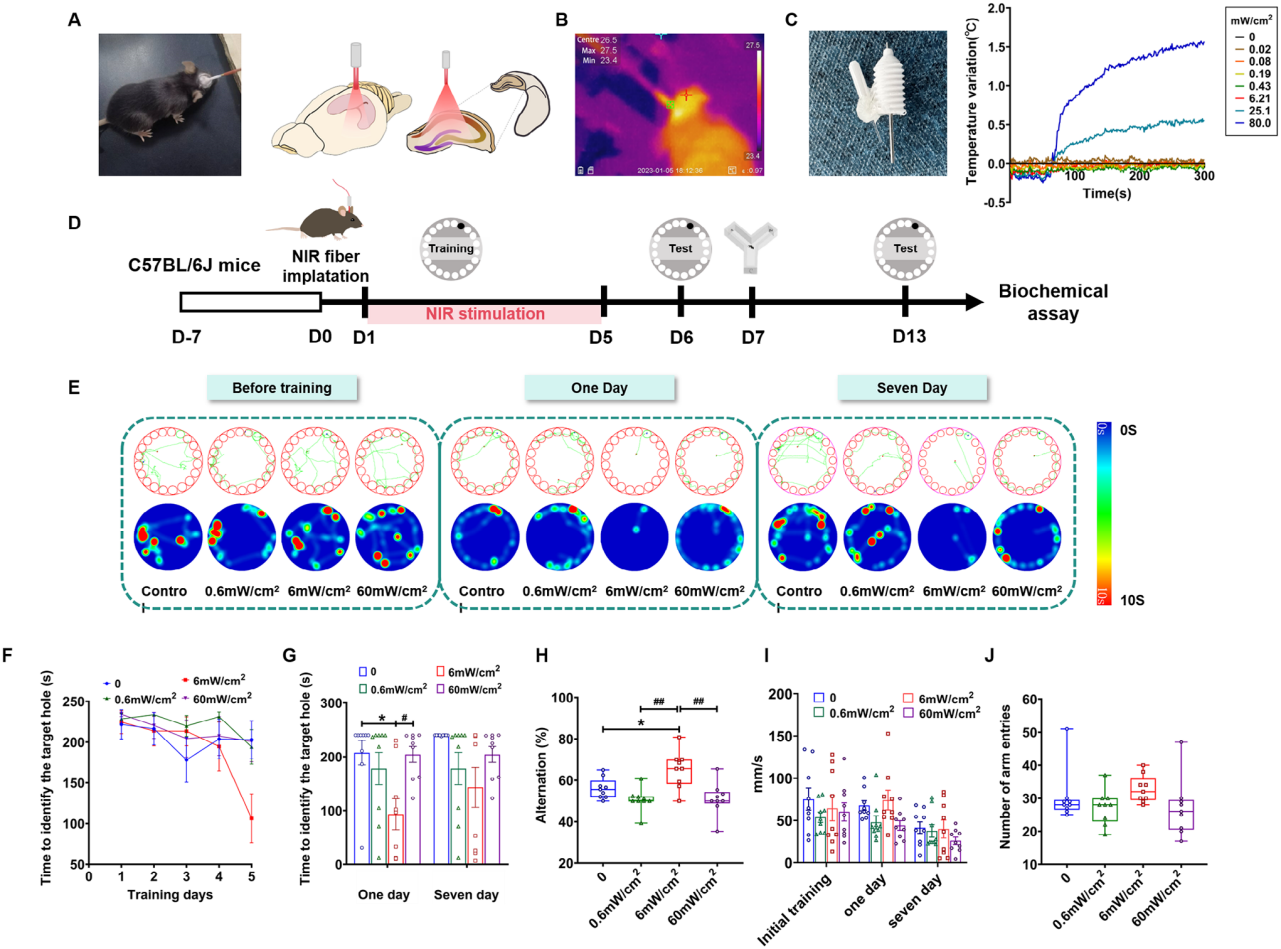
808 nm NIR laser improved the cognitive function of the naïve mice. A) Schematic illustration of 808 nm NIR laser noninvasive irradiating hippocampus by fixing fiber optic cannula on the surface of mouse skull above the hippocampus region. B) The photograph of mouse skull surface temperature measured by a thermal camera. C) Photographs of a self‐designed interbrain tissue thermocouple thermometer and brain temperature measurements during NIR laser irradiation with different irradiance. D) Experimental schematic of 808 nm NIR laser irradiation and behavior tests for naïve mice. E) Typical movement trajectories, F) latency of the training period, and G) latency of the test of mice of the Barnes maze test (interaction: *F*_(3, 32)_ = 0.7674, *p* = 0.5208; test day: *F*_(1, 32)_ = 16.52, *p* = 0.0003; NIR laser irradiance: *F*_(3, 32)_ = 3.901, *p* = 0.0175). H) The proportion of alternation in the four groups during the Y maze test (*F*_(3, 32)_ = 8.480, *p* = 0.0003). I) The average velocity of mice during the Barnes maze test. (interaction: *F*_(6, 64)_ = 0.6490, *p* = 0.6907; test day: *F*_(1.764, 56.44)_ = 10.94, *p* = 0.0002; NIR laser irradiance: *F*_(3, 32)_ = 2.333, *p* = 0.926). J) The total number of arm entry times in the four groups during the Y maze test (*F*_(3, 32)_ = 1.469, *p* = 0.2415). Data in panels (F)–(J) are mean ± s.e.m., *n* = 9 per group. F,G,I) Results were analyzed by two‐way repeated measures ANOVA with Bonferroni's multiple comparisons test and H,J) one‐way repeated measures ANOVA with Bonferroni's multiple comparisons test. Statistically significant differences between 0 and other groups are indicated by asterisks: *p < 0.05. Statistically significant differences between 6 mW cm^−2^ and other groups are indicated by the pound sign: ^#^ *p* < 0.05 and ^##^ *p* < 0.01.

The PBM experiment on the hippocampus of naïve mice was conducted by fixing a fiber optic cannula to the skull surface above the hippocampus (**Figure** **1**A; Figure S1A, Supporting Information). In order to assess the biosafety of 808 nm NIR irradiance for the brain or skin tissue, the deep tissue or surface temperature was measured with a thermal camera (Figure 1B) by using a self‐designed thermocouple thermometer for interbrain tissue (Figure 1C). The temperature measurement in the air was not higher than 25.8 °C (Figure S1B, Supporting Information), in the water was not higher than 27.8 °C (Figure S1C, Supporting Information), and on the mouse skull surface was not higher than 27.5 °C (Figure 1B). The temperature in the hippocampus under various powers (0.02–80 mW cm^−2^) was measured, and the temperature was increased by not more than 2 °C under the highest power of 80 mW cm^−2^ tested (Figure 1C), which was far below “safe temperature” as previously reported.^[^ ^13^ ^]^ Accordingly, four‐gradient irradiation doses (0 as control (CON), 0.6, 6, and 60 mW cm^−2^) were selected to explore the effects of 808 nm NIR laser on the cognitive behavior of the naïve mice, and a schematic setting of the experimental paradigm is shown in Figure 1D. Barnes and Y mazes were applied to test the spatial learning and memory of these naïve mice after irradiation. Mice were irradiated with an 808 nm NIR laser during the training phase of the Barnes maze (days 0–5). As the number of training sessions was increased, all four groups of mice were able to find the target hole faster than on the first day (Figure 1E,F). Indeed, mice exposed to 6 mW cm^−2^ NIR laser spent less time (105.37 s ± standard error of the mean (s.e.m.)) to find the target hole, with a significantly shorter time when compared with the control mice (194.19 s ± s.e.m.) on the first day after training, but this effect was not seen on the seventh day after training (Figure 1G). However, the latency of finding the target hole in mice exposed to 0.6 mW cm^−2^ (178.03 s ± s.e.m.) and 60 mW cm^−2^ (189.69 s ± s.e.m.) NIR laser was not statistically significant compared with that in the control mice. In the Y maze test, the mice that were exposed to the 6 mW cm^−2^ NIR laser showed a significantly higher proportion of alternation entering the arms than the other groups on the first day after training (Figure 1H). All data suggested that 808 nm NIR laser with an irradiance of 6 mW cm^−2^ was preferable for mice to improve spatial learning and memory abilities. Moreover, all groups of mice did not show significant differences in moving speed among in the groups of the Barnes maze and in the total number of arm entry times of the Y maze, suggesting that the latency for mice to find the target hole was not related to their motor ability (Figure 1I,J). To further validate the duration of the PBM, the Barnes and Y mazes were employed on the second and the third day. The results showed that the effect in enhancing the spatial learning and memory for naïve mice undergoing 5 min per day irradiation for 6 days lasted for about 2 days (Figure S2, Supporting Information). Hematoxylin–eosin (H&E) staining was further applied to assess the damage of the brain tissue with 5 days of irradiance. The selected irradiation intensity (0.6, 6, and 60 mW cm^−2^) of the NIR laser all did not cause damage to the brain tissue of mice (Figure S3, Supporting Information). In summary, these experiments demonstrated that the 808 nm NIR laser has significant capability to improve the spatial learning and memory abilities of naïve mice without tissue damage.

### 2.2 808 nm NIR Laser Enhances Multifrequency Oscillations and Theta–Gamma Coupling in the Hippocampal Dentate Gyrus

**Figure 2:**
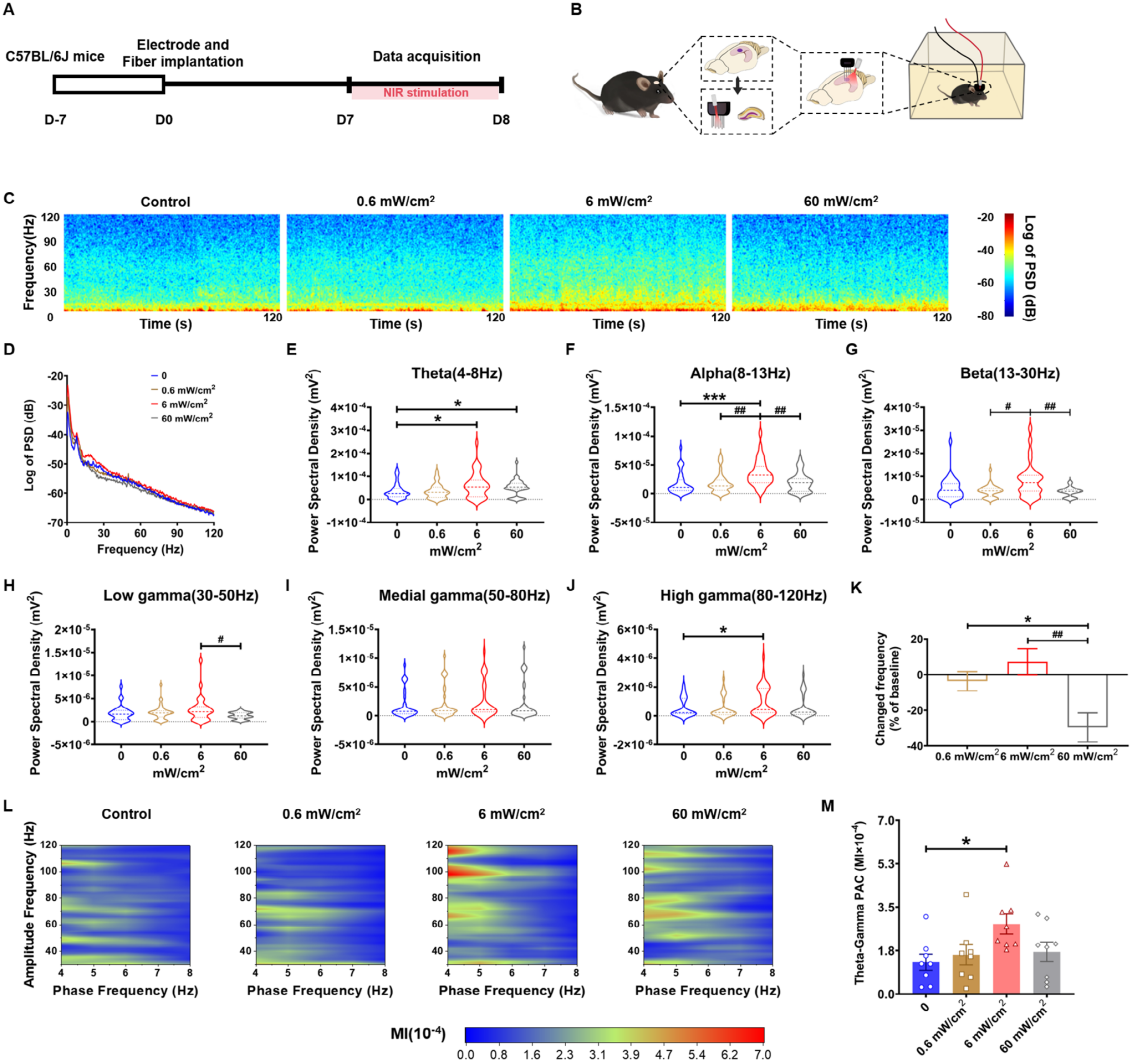
808 nm NIR laser enhanced multifrequency oscillations and theta–gamma coupling in the hippocampal DG of in naïve mice. A) Experimental timeline and schematic diagram showing multichannel recordings in the DG of head‐fixed conscious mice. B) Photographs of the self‐made electrode and fiber core complex. C) Representative heat maps of the power spectrogram in the DG. D) The power spectral density in the DG. E–J The power of oscillations in the DG: E) theta, F) alpha, G) beta, H) low‐frequency gamma, I) medial‐frequency gamma, and J) high‐frequency gamma oscillations in order. K) The rate of change in the number of spikes per second in the DG. The number of spikes per second on 0 mW cm^−2^ of the NIR irradiation was applied as baseline (*F*_(2, 60)_ = 7.132, *p* = 0.0017). L–M) The theta–gamma coupling was enhanced in the DG of the 6 mW cm^−2^ group (*F*_(3, 28)_ = 3.050, *p* = 0.0449). Data in panels (E–K) and (M) are mean ± s.e.m., *n* = 8 per group. Results were analyzed by Kruskal–Wallis ANOVA with E–J) a post hoc Dunn's test and K,M) one‐way repeated measures ANOVA with Bonferroni's multiple comparisons test. Statistically significant differences between 0 and other groups are indicated by asterisks: *p < 0.05 and ***p < 0.001. Statistically significant differences between 6 mW cm^−2^ and other groups are indicated by the pound sign: ^#^ *p* < 0.05 and ^##^ *p* < 0.01.

Hippocampal neuronal network activity is closely related to cognitive function. In this part, we used a self‐made electrode and fiber core complex to study the in vivo local field potential (LFP) in the dentate gyrus (DG) and CA1 of freely moving mice irradiated with 808 nm NIR laser (**Figure** **2**A,B). Compared with nonirradiated mice, the power of neural oscillations in the DG in the irradiated mice was significantly increased with the increase of NIR laser power till it reached an inflection point at the irradiance of 6 mW cm^−2^ and then decreased (Figure S5, Supporting Information). Representative traces of high‐pass‐filtered LFPs of the control and 6 mW cm^−2^ NIR laser irradiation groups are shown in Figure S6 (Supporting Information). However, the power of oscillations in the CA1 region was not significantly increased except alpha oscillation (Figure S7, Supporting Information). Moreover, the number of spikes recorded per second presented in the DG but not CA1 was first increased and then decreased, indicating that 6 mW cm^−2^ was the maximum inflection point as the irradiated power elevated (Figures S8 and S9, Supporting Information). Therefore, NIR irradiance was capable of increasing the LFP and spikes of the DG region.

Further, 6 mW cm^−2^ of NIR irradiation significantly increased the power spectral density in the frequency range from 0 to 120 Hz among the four intensities (Figure 2C,D). For neural oscillation energy, the power of theta (4–8 Hz) (Figure 2E) was significantly higher in the DG only in the group exposed to 6 mW cm^−2^ NIR laser when compared to the control group. When 6 mW cm^−2^ NIR laser irradiance was applied, the power of the alpha oscillation (8–13 Hz) was significantly higher compared to that of the other groups (Figure 2F), but beta oscillation showed no significant difference (13–30 Hz) (Figure 2G). When compared with the control group, the high‐frequency gamma oscillation power (80–120 Hz) of DG in the 6 mW cm^−2^ irradiation group was significantly increased, while the low‐frequency (30–50 Hz) and medium‐frequency (50–80 Hz) gamma oscillations power remained unchanged (Figure 2H,J). Compared with the baseline before the application of NIR laser, the number of spikes per second in the DG exposed to 6 mW cm^−2^ NIR laser had a tendency to rise. However, the number of spikes per second in the DG was significantly lower after 60 mW cm^−2^ irradiation compared with 0.6 and 6 mW cm^−2^ irradiation (Figure 2K).

Gamma oscillation is a key rhythmic neuronal activity that underlies cognitive and affective behavior.^[^ ^14^ ^]^ Hierarchical cross‐frequency phase–amplitude coupling (PAC) of neuronal oscillations, in which the phase of slower oscillations modulates the amplitude of faster oscillations within a region or between different regions, has been proposed as a general mechanism involved in memory information encoding, storage, and retrieval.^[^ ^15^ ^]^ Accordingly, we further calculated the PAC between theta (slow wave) and gamma (fast wave), as an indicator of the level of population neuronal activity integration, and estimated the effect of NIR laser on the PAC between low‐frequency and high‐frequency oscillations.^[^ ^16^ ^]^ The results showed that the PAC of mice receiving 6 mW cm^−2^ irradiation was significantly increased compared with the other groups (0, 0.6, and 60 mW cm^−2^) (Figure 2L,M). Given the existence of a coupling mechanism between gamma and theta oscillations,^[^ ^17^ ^]^ the increase in gamma oscillation energy in the DG may be at least partly due to stronger theta–gamma PAC, leading to a further increase in neuronal network excitability. These results revealed that 808 nm NIR laser may improve the cognitive function of naïve mice by enhancing multifrequency oscillations and theta–gamma coupling in the hippocampal DG.

### 2.3 808 nm NIR Laser Specifically Activates Neurons in the Hippocampal DG

**Figure 3:**
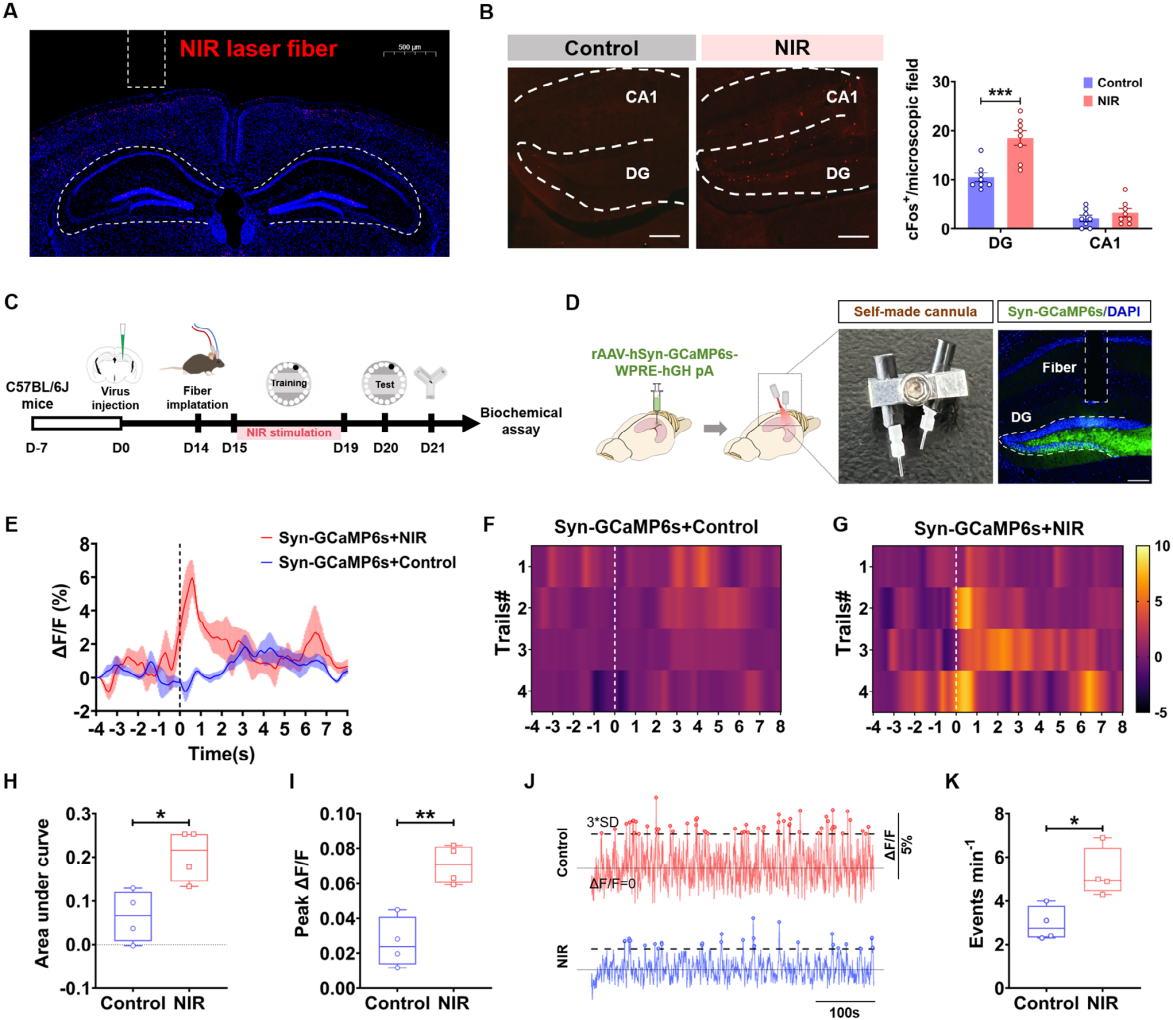
808 nm NIR laser specifically activated neurons in the DG of the hippocampus. A) Representative immunofluorescence images of c‐Fos (red) and DAPI (blue) staining in CA1 and DG of mice, showing the implantation site of the fiber core (scale bar, 500 µm). B) Left: representative immunofluorescence images of c‐Fos (red) staining in CA1 and DG of mice (scale bar, 200 µm) of the control and NIR groups. Right: quantitative data of the number of c‐Fos positive cell in the CA1 and DG of mice from the two groups (interaction: *F*_(1, 28)_ = 11.29, *p* = 0.0023; brain region: *F*_(1, 28)_ = 133.4, *p* < 0.0001; and treatment: *F*_(1, 28)_ = 19.89, *p* < 0.0001). C) Experimental schematic of 808 nm NIR laser irradiation, behavior tests, and fiber photometry. D) Left: schematic presentation of the viral injections. Right: picture of self‐made cannula and representative images of rAAV‐hSyn‐GGaMP6s expression in the DG (scale bar, 200 µm). E–G) Average fluorescent signals and heat maps of GCaMP6s expressed in DG neurons evoked by entering the target hole. H–I) Quantification of H) GCaMP6s fluorescent signals’ AUC (*t*_(6)_ = 3.338, *p* < 0.05) and I) peak (*t*_(6)_ = 4.936, *p* < 0.01) in DG neurons. J) Representative traces of population Ca^2+^ activity in DG neurons during Y maze (8 min). K) Events of population Ca^2+^ activity in DG (*t*_(6)_ = 3.337, *p* < 0.05). Data in panels (B), (E), and (H–K) are mean ± s.e.m., B) *n* = 8 per group for panels (B) and *n* = 4 per group for panels (E–K). Results were analyzed by B) two‐way repeated measures ANOVA with Bonferroni's multiple comparisons test and H–K) two‐tailed paired Student's *t*‐test. Statistically significant differences between control and NIR laser groups are indicated by asterisks: *p < 0.05, **p < 0.01, and ***p < 0.001.

In order to further explore for why NIR laser significantly enhanced neuronal network activity in the DG and cognitive function in naïve mice, the neuronal activation of DG and CA1 with c‐Fos immunofluorescent staining was conducted (**Figure** **3**A). The 6 mW cm^−2^ 808 nm NIR laser activated more c‐Fos positive neurons in the DG, but not in CA1 and the nonlaser irradiation (Figure 3B), suggesting significant neuronal activation in DG during NIR laser irradiation. In order to further study the effect of NIR laser stimulation on neuronal activity in the DG region, rAAV‐hSyn‐GCaMP6s was injected into the DG of the naïve mice. Then, a self‐made fiber optic cannula was implanted in the injection site and, therefore, NIR stimulation and calcium signal recording both can be done at the same time. The Barnes maze paradigm was used to test the spatial learning ability on the first day after cannula implantation, and the NIR stimulation was conducted only during the training process (Figure 3C). Ca^2+^ signal recording by fiber photometry was performed on the last day during the Barnes maze test. The results showed that mice exposed to NIR laser irradiation during training had significantly enhanced Ca^2+^ signal intensity in the DG when they successfully entered the target hole during test (Figure 3D–I). On the next day of the Barnes maze test, the mice underwent the Y maze test and Ca^2+^ signal recording simultaneously. Surprisingly, the frequency of Ca^2+^ signal events in hippocampal DG neurons of the NIR group was higher than that of control group during the Y maze test (Figure 3J,K). These results indicated that the NIR laser activates cognitive‐behavior‐related neurons, particularly in the DG of the hippocampus.

### 2.4 808 nm NIR Laser Activated Both Excitatory and Inhibitory Neurons

**Figure 4:**
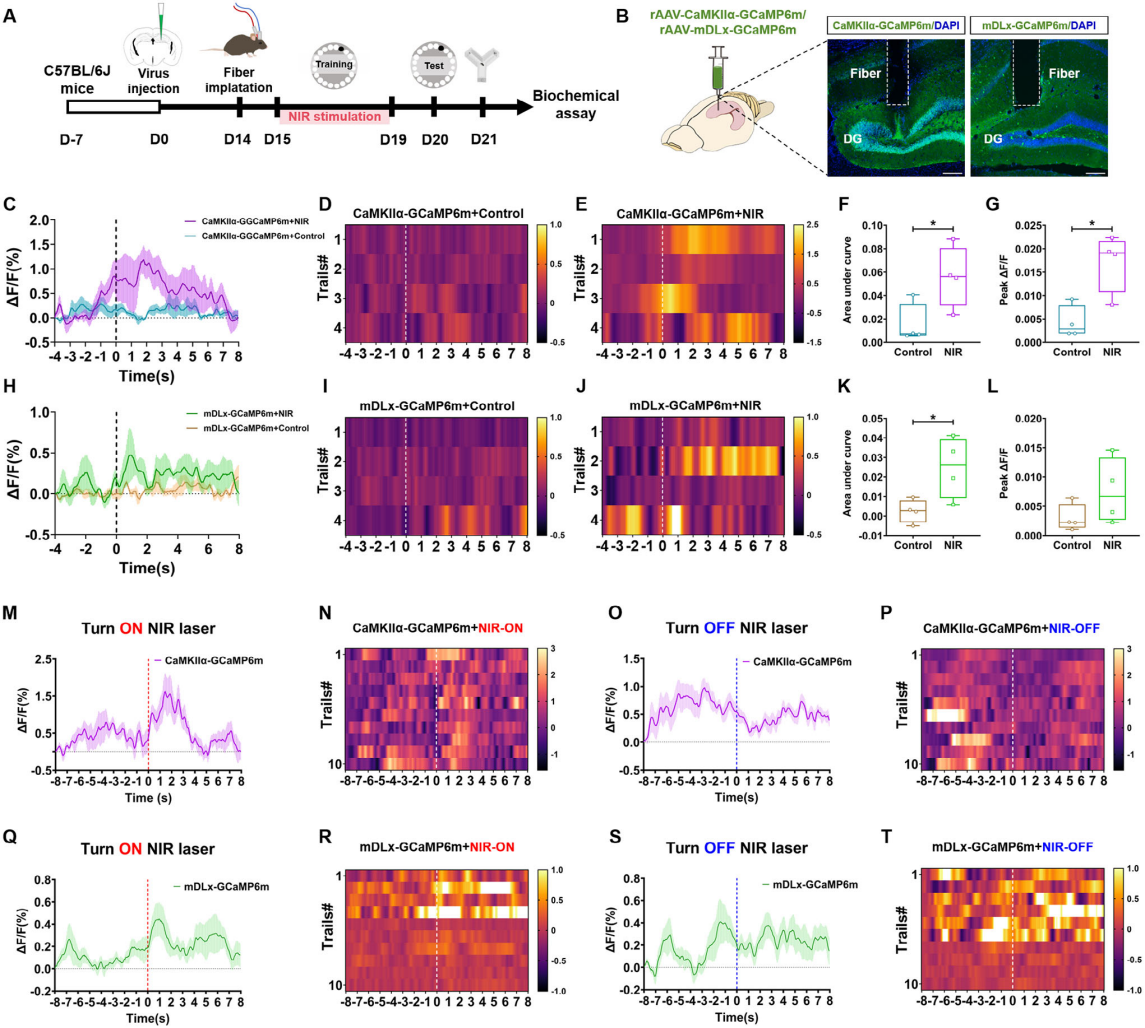
Identification of neuronal types activated by 808 nm NIR laser. A) Experimental schematic of 808 nm NIR laser irradiation, behavior tests, and fiber photometry. B) Schematic presentation of the viral injection, and representative images of rAAV‐CaMKIIα‐GCaMP6m and rAAV‐mDLx‐GCaMP6m expressions in the DG (scale bar, 200 µm). C–G) Average fluorescent Ca^2+^ signals and heat maps in DG excitatory neurons evoked by entering the target hole. Quantification of fluorescent F) Ca^2+^ signals’ AUC (*t*_(6)_ = 2.602, *p* < 0.05) and G) peak (*t*_(6)_ = 3.604, *p* < 0.05) in DG excitatory neurons. H–L) Average fluorescent Ca^2+^ signals and heat maps in DG inhibitory neurons evoked by entering the target hole. Quantification of fluorescent K) Ca^2+^ signals’ AUC (*t*_(6)_ = 1.835, *p* < 0.05) and L) peak (*t*_(6)_ = 1.508, *p* = 0.1823) in DG inhibitory neurons. M–P) Average fluorescent Ca^2+^ signals and heat maps in DG excitatory neurons evoked by turning on/off NIR laser. Q–T) Average fluorescent Ca^2+^ signals and heat maps in DG inhibitory neurons evoked by turning on/off NIR laser. Data in panels (C),(F–H), (K–M), (O), (Q), and (S) are mean ± s.e.m., *n* = 4 per group for panels (C–L), *n* = 8 per group for panels (M–T). F,G,K,L) Results were analyzed by two tailed paired Student's t‐test. Statistically significant differences between control and NIR laser groups are indicated by asterisks: *p < 0.05.

To further investigate the specificity of neuronal activation in the DG region by NIR laser stimulation, we aimed to distinguish the types of activated neurons. The mice were divided into two groups and injected with rAAV‐CaMKIIα‐GCaMP6m (expression in excitatory neurons) and rAAV‐mDLx‐GCaMP6m (expression in inhibitory neuron) into the DG, respectively. The Barnes maze paradigm was used to test the spatial learning ability on the first day after cannula implantation, and the NIR stimulation was conducted only during the training process (**Figure** **4**A). Ca^2+^ signal recording by fiber photometry was performed on the last day during the Barnes maze test. Compared with the non‐NIR groups, the Ca^2+^ activity of the NIR‐irradiated groups was significantly increased when they entered the target hole (Figure 4B–L). Thus, NIR‐laser‐induced neuronal Ca^2+^ activity was not selective of the excitatory and inhibitory neurons of the DG related to spatial memory processing. The Y maze test was performed on the next day. These data did not show any statistical differences (Figure S10, Supporting Information).

To affirm the correlation of NIR irradiation and Ca^2+^ activity, we further investigated immediate DG neuronal Ca^2+^ activity when turning on and off the NIR laser during free movement. The Ca^2+^ activity, either in the rAAV‐CaMKIIα‐GCaMP6m group or the rAAV‐mDLx‐GCaMP6m group, was significantly increased with the NIR laser on but soon decreased as the laser was turned off (Figure 4M–T). More importantly, NIR‐induced neuronal Ca^2+^ activity showed nonselectivity between excitatory and inhibitory neurons. Additionally, we further used vGlut2‐Cre mice as representation to affirm the effect of NIR laser irradiation on the glutaminergic neuronal Ca^2+^ activity. After rAAV‐hSyn‐DIO‐GCaMP6f injection, the NIR groups had higher DG's neuronal Ca^2+^ activity than the control group during the point which mice entered the target hole (Figure S11A–E, Supporting Information). The immediate DG's neuronal Ca^2+^ activity when turning on and off the NIR laser was also conducted and obtained similar results to the above (Figure S11F–I, Supporting Information). These findings indicated that NIR laser stimulation has the ability to rapidly and transiently modulate the activity of both excitatory and inhibitory neurons in the DG region. To verify the accuracy of the above results, ontological fluorescence interference of brain tissue was detected by fiber photometry to record signal of virus‐free DG region under NIR laser irradiation and the recording signal was close to zero (Figure S11J–M, Supporting Information), which indicated that the changes of DG's neuronal Ca^2+^ activity observed were solely attributed to the effects of NIR laser irradiation.

### 2.5 High‐Density Cell‐Structure‐Mediated NIR Laser‐Specific Activation of Neurons in the Hippocampal DG

**Figure 5:**
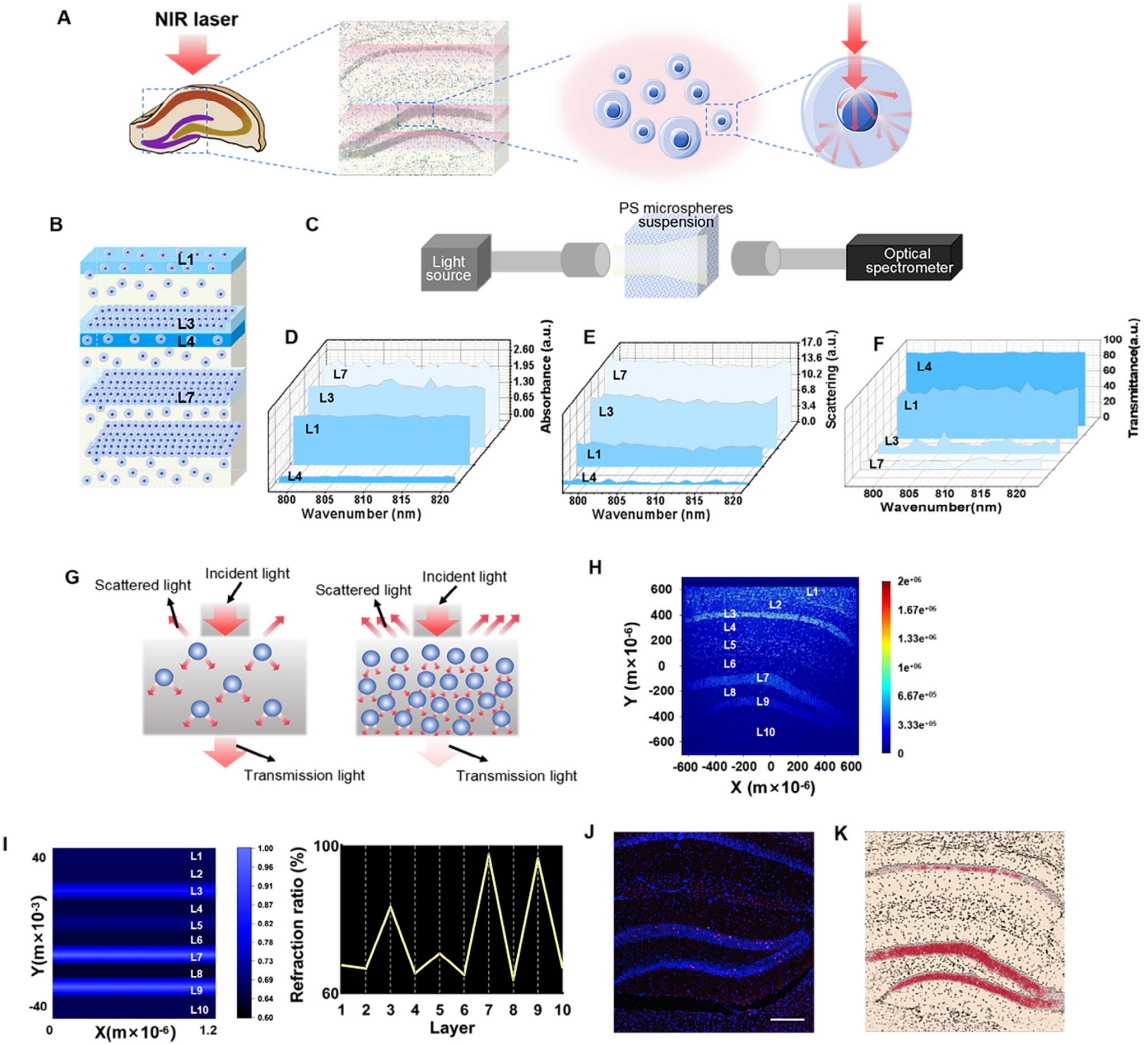
High‐density cell‐structure‐mediated NIR laser specific activation of neurons in the DG. A) Schematic representation of the multilayer “tissue–cell–organelle” system in the hippocampus of mice. B) Schematic representation of cell density stratification in the mouse hippocampus region in vitro. C–F) Schematic diagram of the optical detected experiment for the PS microsphere suspensions. D–F) The D) absorbance, E) scattering, and F) transmittance spectra of PS microspheres suspension with four typical concentrations according to the cell density of hippocampus from 800 to 820 nm. G) Schematic representation of the forward scattering of the NIR laser. H) The FDTD simulation on nuclear scattering of 808 nm NIR laser penetrated into hippocampus tissue. I) The FDTD simulation on refractive index difference of 808 nm NIR laser penetrated into hippocampus tissue. J) The typical immunofluorescence images of c‐Fos (red) and DAPI (blue) staining in CA1 and DG of mice. K) The schematic diagram of 808 nm NIR photodeposition penetrated in hippocampus tissue.

In our study, we fixed the NIR fiber vertically on the skull surface to irradiate the below CA1 and DG regions. As previously reported, due to the micrometer and sub‐micrometer organelles with different dielectric constants, such as the nucleus and mitochondria among the multilayer “tissue‐cell‐organelle” system, the incident NIR laser resulted in scattering, reflection and absorption in the tissue^[^ ^18^ ^]^ (**Figure** **5**A). Noticeably, histological staining of the hippocampus showed that the CA1 and DG regions both had special anatomical structures: densely packed cell layer; the CA1 has one while the DG regions have two densely packed cell layers (Figure S12B, Supporting Information). NIR‐activated neurons were more located on the densely packed cell layers in both DG and CA1 regions. Therefore, we speculated that the enhanced DG's neuronal activity might be attributed to the denser cell layers in the DG region than the CA1 region, which were conducive to generate more local scattering and promoted the NIR laser residing in the tissues, forming local energy deposition. To confirm this hypothesis, we simulated the 808 nm NIR laser energy distribution across the hippocampus using microspheres optical experiment as well as the finite difference time domain (FDTD) method with the two main factors: local scattering effect and local refractive index.

It is reported that the strong scattering effect of biological tissues is mainly due to the inhomogeneity of refractive index at the cellular scale, and the nucleus contributes the most to the lateral scattering (80°–100°) among the various scattering.^[^ ^19^ ^]^ Therefore, the scattering effect induced by nuclei may be the main factor determining the local energy deposition of NIR light in biological tissues. To confirm this, we conducted an in vitro optical validation test using 5 µm polystyrene (PS) microsphere serving as a nucleus. In order to simulate the real situation, we divided the 4',6‐Diamidino‐2‐phenylindole dihydrochloride (DAPI)‐stained hippocampus into ten layers according to the DAPI densities from top to bottom (Figure 5B). The density of DAPI fluorescent spots was measured with software, and the 3D density of each layer was calculated as 0.323, 0.028, 13.984, 0.010, 0.189, 0.027,46.424, 0.199, 28.545, and 0.066 (Figure S12, Supporting Information). Then, four typical layers (L4, L1, L3, and L7, sorted by density from the smallest to the largest) were selected (Figure S13, Supporting Information) to conduct the optical measurement including reflection, absorption, and transmittance using a fiber optic spectrometer (Figure 5C). As the concentration of PS microspheres increased, the absorption and scattering of the suspension gradually strengthened, but the transmittance gradually decreased. This was detected in 550–900 nm of visible–near‐infrared (vis–NIR) waveband (Figure S14, Supporting Information). Furthermore, the PS microspheres’ suspension with high concentration (46.42 mg mL^−1^, calculated from L7) near 808 nm NIR laser had higher light absorptivity (Figure 5D), lower light scattering (Figure 5E) and lower light transmittance (Figure 5F) than that of PS microspheres’ suspension with low concentration (0.01 mg mL^−1^, calculated from L4). Therefore, these results suggest that a high‐density nucleus is more likely to promote NIR laser scattering locally and retain NIR laser in the medium than a low‐density nucleus (Figure 5G). Moreover, we used FDTD to create further simulations according to the DAPI‐stained high‐resolution fluorescence photo. The simulated light energy distribution image and concluded that 808 nm NIR laser does show a high absorption in the high‐density cell layers (L3, L7, and L9) (Figure 5H).

As reported, the nucleus has a refractive index of 1.42 and accounts for 10% of the total cell volume, and water is the most abundant compound in living cells, accounting for about 80–90% of the fresh cell weight, and extracellular matrix refractive index is 1.37 on average.^[^ ^20^ ^]^ Therefore, the refractive index of the hippocampus is inversely proportional to the density of cells. Based on the difference of the refractive index for each layer, we used the FDTD method to further simulate the 808 nm NIR laser propagating across hippocampal CA1 and DG regions. The results concluded that 808 nm NIR laser tended to deposit in dense cell layers (L3, L7, and L9) with a relatively low refractive index, especially at the junctional interface from low refractive index to high refractive index. This may be another major reason for the localization of light in high‐density cell layers and energy deposition (Figure 5I). These results demonstrated that high local scattering and low refraction of the high‐dense cell structure of the DG region may be an important reason for local NIR energy deposition and neuron activation (Figure 5J,K). More importantly, 1064 nm NIR laser was also used to remotely irradiate the hippocampus, and the results showed that more cells in the DG region were also activated by the laser (Figure S15, Supporting Information), which demonstrated that the local energy deposition mediated by high‐density cell structure might also be applicable for NIR.

### 2.6 808 nm NIR Laser Activates Axonogenesis and Oxidative Phosphorylation Pathways in DG

To analyze cellular heterogeneity and explore the changes of key molecules and pathways in neuronal cells in the hippocampal DG region of mice after 5 day 808 nm NIR laser irradiation, we performed drop‐based single‐cell RNA sequencing of cells in the hippocampal DG of mice.

**Figure 6:**
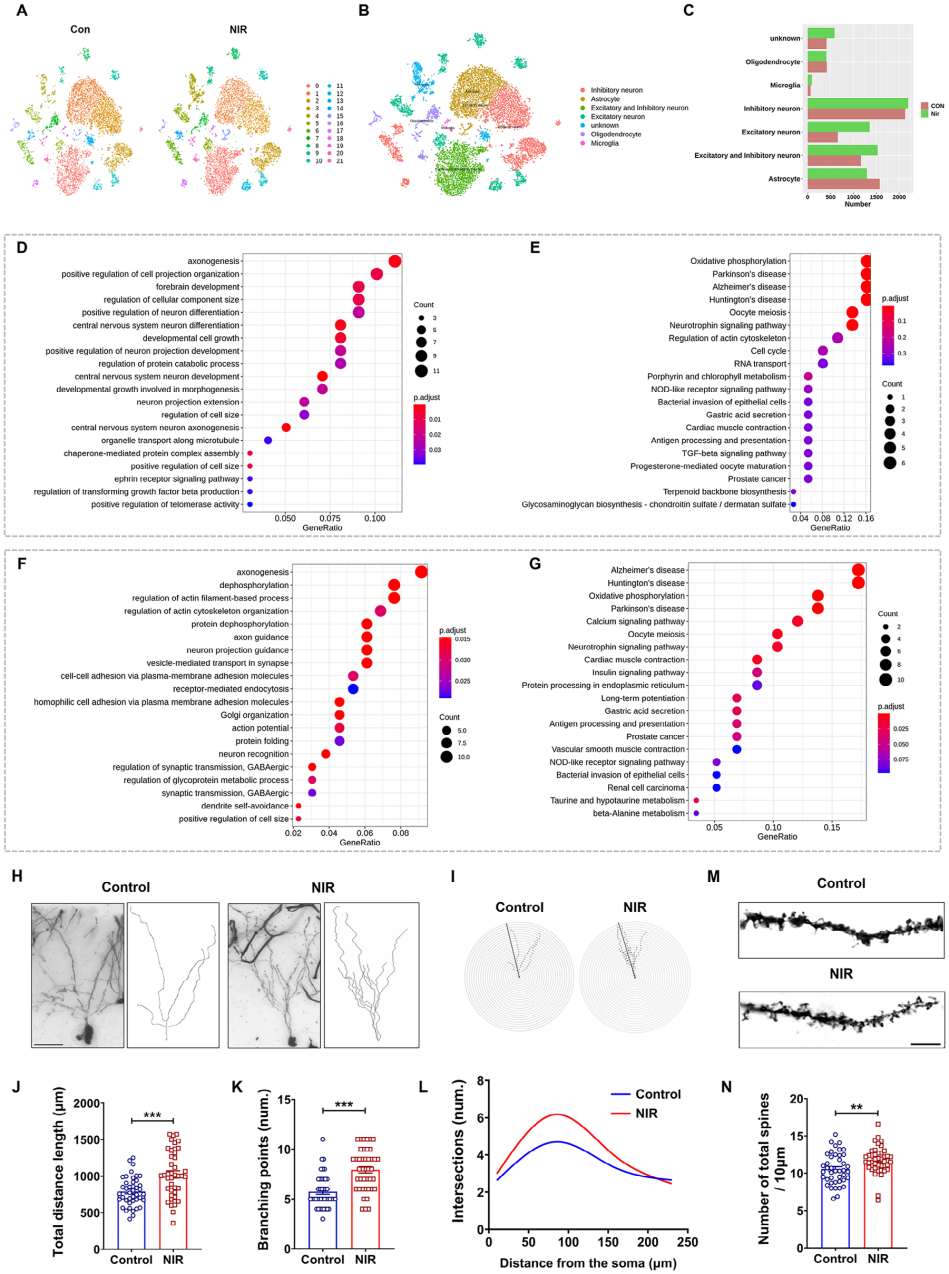
808 nm NIR laser activates axonogenesis and oxidative phosphorylation pathways in DG. A) Visualization of t‐SNE for all clusters (samples are presented separately, 16 216 cells). Dots, individual cells; colors, neuron clusters. B) Results of single‐cell type annotation based on marker genes. C) Statistical plots of cell numbers of different samples from each cluster. D,E) Bubble plot of GO and KEGG enrichment of marker genes of excitatory neuron. F,G) Bubble plot of GO and KEGG enrichment of marker genes of inhibitory neuron. H) The representative Golgi staining was performed to visualize the dendritic morphology in DG neurons. Scale bar  =  50 µm. I–L) A schematic representation of the Sholl analysis method was used to assess the dendritic branching of hippocampal neurons. A series of concentric circles centered on the cell body at consecutive intervals of 10 µm were drawn to obtain the number of intersections/branching points with the cell body as a function of distance. M) Representative spines of axons in the DG of the hippocampus. Scale bar  =  5 µm. N) The number of spines per 10 µm axon was quantified in both groups of mice. Data in panels (J–L) and (N) are mean ± s.e.m., *n* = 42 neurons (7 neurons per mouse) of the control and NIR groups. J,K,N) Data were analyzed by two‐tailed paired Student's *t*‐test. Statistically significant differences between control and NIR groups are indicated by asterisks: **p < 0.01 and ***p < 0.001.

After stringent quality control and filtering, 16216 single‐cell transcriptomes were obtained and were clustered into 21 clusters by the *t*‐distributed stochastic neighbor embedding (t‐SNE) reduction (**Figure** **6**A; Figure S16A, Supporting Information). Differential gene expression analysis identified cluster‐specific marker genes (Figure S16B, Supporting Information). Combined with differentially expressed genes (DEGs), several known hippocampal cell‐type‐specific markers were utilized to annotate cell populations (Figure 6B; Figure S16C, Supporting Information). In addition, the number of neurons was increased after 808 nm NIR irradiation (Figure 6C; Figure S16D, Supporting Information).

Furthermore, we analyzed the differentially expressed genes between the excitatory neurons (Figure S17A, Supporting Information) and inhibitory neurons (Figure S17B, Supporting Information) of the NIR and CON groups. The expressions of the Actb and cytochrome C oxidase‐related genes (Cox6c, Cox6c) were significantly increased in the NIR group. Actb is closely related to the process of axonogenesis by participating in cytoskeleton construction, cell migration and signaling, and providing the driving force for cell movement. These functions are essential for the generation and maintenance of nerve cell axons.^[^ ^21^ ^]^ Moreover, the main mechanism of NIR laser action on cells was to induce CCO in cells. Cells absorb optical energy, causing the redox state of CCO to change from a rest state to an excited state, which then activates the mitochondrial electron‐transport chain. Subsequently, downstream signaling pathways were activated to protect neurons, promote neuronal proliferation, and form new synapses for better memory formation.^[^ ^4^ ^]^ Therefore, the results of differential gene analysis focused on axon and CCO, reflecting the mechanism of neuronal activation by 808 nm NIR laser.

In Gene Set Enrichment Analysis (GSEA), each cluster was compared with other clusters to explore the functional heterogeneity of different cluster cells. Without differential analysis, sphingolipid signaling pathway and synaptic vesicle cycle synthesis were significantly upregulated in the excitatory neurons of the NIR group compared with the CON group. Activation of both pathways was associated with axon growth and function (Figure S17C, Supporting Information).

The marker genes of each cluster represent the characteristic expressed genes of the cluster, but a single gene may not reflect the characteristics of the cluster as a whole. Therefore, Kyoto Encyclopedia of Genes and Genomes (KEGG) and Gene Ontology (GO) enrichment analyses were performed on the marker genes of each cluster. The marker genes (log_2_^FC^ > 0, *p* < 0.05) that were significantly more highly expressed in each cluster were compared with the other clusters. In excitatory neurons, the top 5 or top 20 GO terms and differentially expressed genes were mainly enriched in axonogenesis (Figure 6D; Figure S18A, Supporting Information). Among the top 5 or 20 KEGG pathways, the differentially expressed genes were mainly enriched in the oxidative phosphorylation pathway (Figure 6E; Figure S18B, Supporting Information). In inhibitory neurons, the top 5 or top 20 GO terms, differentially expressed genes were mainly enriched in axonogenesis, dephosphorylation, regulation of actin filament‐based process, and regulation of actin cytoskeleton organization (Figure 6F; Figure S18C, Supporting Information). Among the top 5 or 20 KEGG pathways, the differentially expressed genes were mainly enriched in the oxidative phosphorylation pathway (Figure 6G; Figure S18D, Supporting Information). Therefore, the mechanism of neuronal activation by NIR laser is related to axonogenesis, which is achieved through the regulation of oxidative phosphorylation.

Further analysis of differential genes showed that CCO subunit genes, heat shock protein genes, and mitochondrial GTPase genes were significantly increased in neurons after NIR irradiation (Figure S18E, Supporting Information), indicating that 808 nm NIR activates CCO involved in the mitochondrial electron‐transport chain.

Given that the high enrichment of differentially expressed genes for axonogenesis in single‐cell sequencing, we further performed Golgi staining to explore whether the usage of an 808 nm NIR laser enhanced neuronal activity in the DG. This would be achieved through altering the structure of axon and dendritic spines. The morphology and axon structure diagram of representative neurons in the DG are shown in Figure 6H. Sholl analysis showed that NIR laser significantly increased the length of dendrites, the number of branching points, and dendritic intersections related to controls without treatment (Figure 6I–L). There was also a significant difference in the number of spines per 10 µm of dendrites after NIR laser treatment (Figure 6M,N).

### 2.7 808 nm NIR Laser Rescued Cognitive Dysfunction and Improved the Energy of Multiple Frequency and Theta–Gamma Coupling of DG Neurons

**Figure 7:**
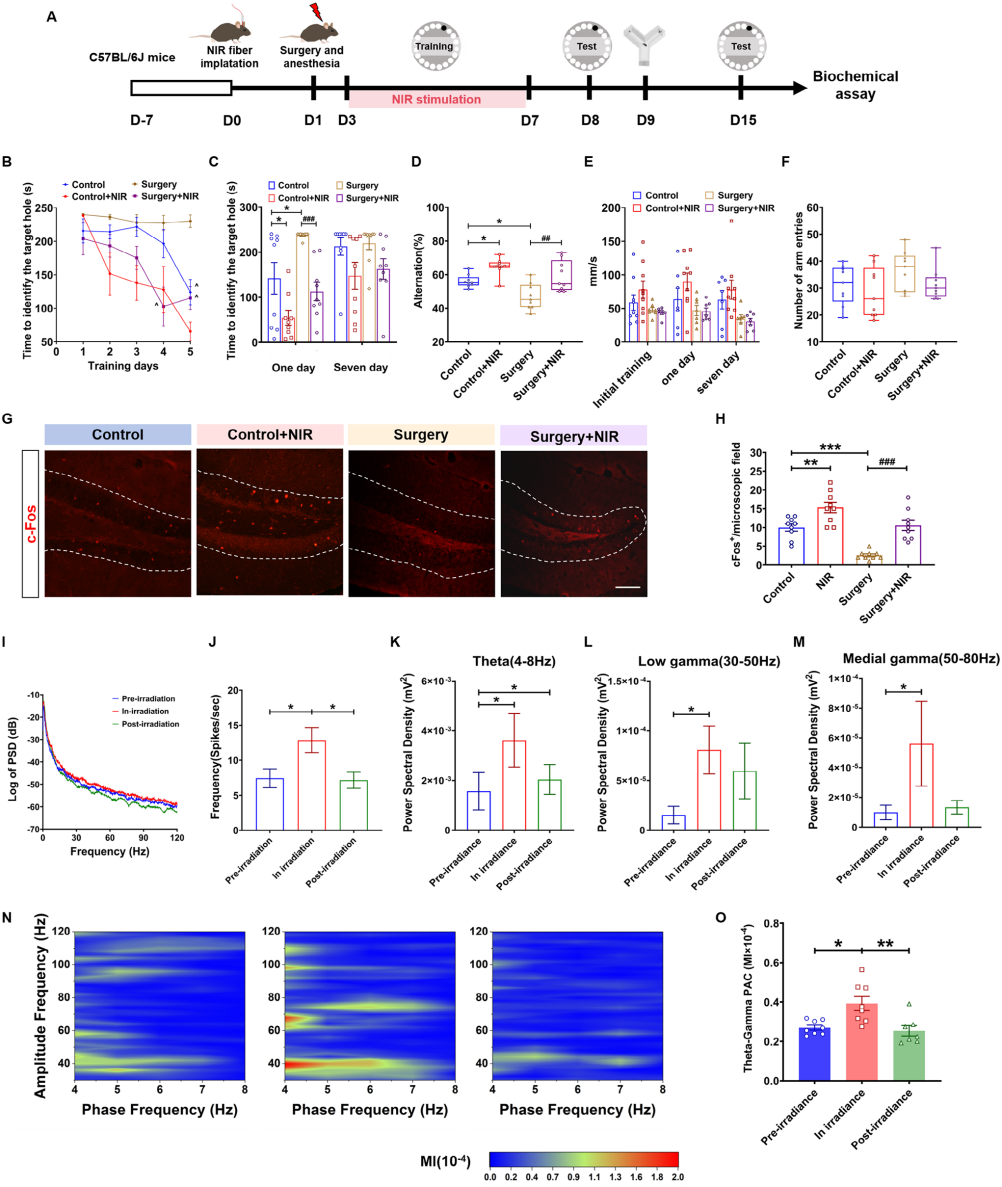
808 nm NIR laser rescued cognitive dysfunction and improved the energy of multiple frequency and theta–gamma coupling of DG of POCD model mice. A) Experimental schematic of 808 nm NIR laser irradiation and behavior tests (POCD model). B) Identify latency of the training period and C) identify latency of the test of mice during the Barnes maze test (interaction: *F*_(3, 32)_ = 2.766, *p* = 0.0578; test day: *F*_(1, 32)_ = 11.48, *p*= 0.0019; and treatment: *F*_(3, 32)_ = 10.71, *p* < 0.0001). D) The proportion of alternation in the four groups during the Y maze test (*F*_(3, 32)_ = 11.06, *p* < 0.0001). E) The average velocity of mice during the Barnes maze test (interaction: *F*_(6, 80)_ = 0.2001, *p* = 0.9758; test day: *F*_(2, 80)_ = 0.7819, *p* = 0.4610; and treatment: *F*_(3, 80)_ = 9.747, *p* < 0.0001). F) The total number of arm entry times in the four groups during the Y maze test (*F*_(3, 32)_ = 1.773, *p* = 0.1720). G) Representative immunofluorescence images of c‐Fos (red) staining in the DG of mice (scale bar, 100 µm). H) Quantitative data of the number of c‐Fos positive cell in the DG of mice (*F*_(3, 32)_ = 23.29, *p* < 0.0001). I) Representative line chart of the corresponding power spectral density pre‐, in‐, and post‐NIR irradiance recording from the DG. J) The rate of change in the number of spikes per second pre‐, in‐, and post‐NIR irradiance recording from the DG (*F*_(2, 27)_ = 5.004, *p* = 0.0142). K–M) The power of oscillations pre‐, in‐, and post‐NIR irradiance recording from the DG: K) theta, L) low‐frequency gamma, and M) medial‐frequency gamma oscillations in order. N,O) Representative heat maps of N) theta–gamma coupling and O) the corresponding theta–gamma PAC, pre‐, in‐, and post‐NIR irradiance in the DG (*F*_(2, 20)_ = 8.068, *p* = 0.0027). Data in panels (B–F), (H), (J–M), and (O) are mean ± s.e.m., *n* = 8–9 per group. Results were analyzed by B,C,E) two‐way repeated measures ANOVA with Bonferroni's multiple comparisons test, D,F,H,J,O) one‐way repeated measures ANOVA with Bonferroni's multiple comparisons test, K–M) and Kruskal–Wallis ANOVA with a posthoc Dunn's test. Statistically significant differences between control and other groups are indicated by asterisks: ^*p* < 0.05, *p < 0.05, **p < 0.01, and ***p < 0.001. Statistically significant differences between surgery and other groups are indicated by the pound sign: ^#^ *p* < 0.05, ^##^ *p* < 0.01, and ^###^ *p* < 0.001.

Based on the 808 nm NIR laser's enhancement of neuronal activity for the DG in naïve mice, we further investigated the effect of PBM on the cognitive behavior in a POCD model.^[^ ^22^ ^]^ The detailed behavioral paradigm is shown in **Figure** **7**A. The Barnes maze test was first conducted to evaluate the cognitive behavior, of which the NIR laser was used only during the training block. As the training times increased, the time for all groups of mice to find the target hole in the Barnes maze was significantly decreased compared to their initial training time (Figure 7B; Figure S19A, Supporting Information). Furthermore, the time taken for the surgery mice without laser irradiation to find the target hole was far longer (237.6 s ± s.e.m.) than that of the control mice (141.94 s ± s.e.m.) without laser irradiation on the first day after training, suggesting that the mice POCD model was successfully established. Moreover, the control mice exposed to NIR laser took less time (54.6 s ± s.e.m.) to find the target hole, performing a significant shortening compared with their counterparts without treatments (141.9 s ± s.e.m.; *p* < 0.05) on the first day after training, but this effect was not seen on the seventh day after training. Similarly, the surgery mice exposed to NIR laser needed much less time (112.61 s ± s.e.m.) to find the target hole when compared with the surgery mice (237.6 s ± s.e.m.; *p* < 0.05) on the first day after training, but this effect was not significant on the seventh day after training (Figure 7C). In the Y maze test, NIR laser irradiation significantly increased the proportion of alternation of the POCD mice (control: 56.1%, control + NIR: 64.8%, surgery: 46.9%, and surgery + NIR: 59.2%) (Figure 7D). In addition, there was no significant difference in the movement speed during the Barnes maze test as well as the total number of arm entry of the Y maze between the NIR irradiation and non‐NIR irradiation (Figure 7E,F). Collectively, these results indicated that PBM can improve the cognition of naïve mice and showed therapeutic effects in improving cognition in POCD model mice.

To further affirm the neuronal activation of the DG region, the DG neurons were harvested and subjected to immunofluorescence staining after the behavioral test. The results showed that the fluorescence intensity of c‐Fos in the DG of the surgery group was significantly lower than that of the control group. After NIR irradiation, the fluorescence intensity of c‐Fos in the DG of the surgery group was significantly increased (control: 10 cells per microscopic field, control + NIR: 15.3 cells per microscopic field, surgery: 2.56 cells per microscopic field, and surgery + NIR: 10.56 cells per microscopic field), indicating that NIR irradiation triggered significant neuronal activation in the DG, which may be related to the improvement of cognitive ability (Figure 7G,H).

The 808 nm NIR laser reversed the LPS‐induced cognitive dysfunction without the motor movement change. The neurons in the DG regions were significantly activated after NIR laser irradiation (Figure S20, Supporting Information). The tumour necrosis factor‐α (TNF‐α) content in the DG region in the two disease models after NIR laser irradiation was not statistically different, indicating that 808 nm NIR laser improved cognitive function. However, this was not due to reducing neuroinflammation (Figure S21, Supporting Information).

In vivo electrophysiology recording was applied in the DG while freely moving under NIR laser irradiation (Figure S19B, Supporting Information). The real‐time recording LFP signals and spikes were statistically analyzed during the 2 min before, during, and after NIR laser irradiation. The power spectral density in the frequency range from 0 to 120 Hz was significantly increased during NIR irradiation (Figure 7I; Figure S19C, Supporting Information). The number of spikes per second in the DG was significantly higher in mice when exposed to NIR laser irradiation than pre‐ and post irradiation (Figure 7J). For neural oscillation energy, the power of theta (4–8 Hz) (Figure 7K) was significantly increased in the DG irradiated with NIR laser compared with that before and after irradiation, while there was no significant difference in alpha oscillations (8–13 Hz) and beta oscillations (13–30 Hz) (Figure S19D,E, Supporting Information). Moreover, in addition to high‐frequency gamma oscillation (80–120 Hz), the power of low‐frequency gamma oscillations (30–50 Hz) and medium‐frequency gamma oscillations (50–80 Hz) in the DG was also increased significantly during NIR laser irradiation (Figure 7L,M; Figure S19F, Supporting Information).

Moreover, we further calculated PAC between theta (slow wave) and gamma (fast wave), and estimated the effect of NIR laser on PAC between low‐frequency and high‐frequency oscillation in the POCD model. The results showed that the PAC was significantly higher during NIR laser irradiation than pre or post irradiations (Figure 7N,O) These data suggested that 808 nm NIR laser also held the ability to enhance the multifrequency oscillations and theta–gamma coupling of hippocampal DG in the POCD model of mice.

## 3 Discussion

**Figure 8:**
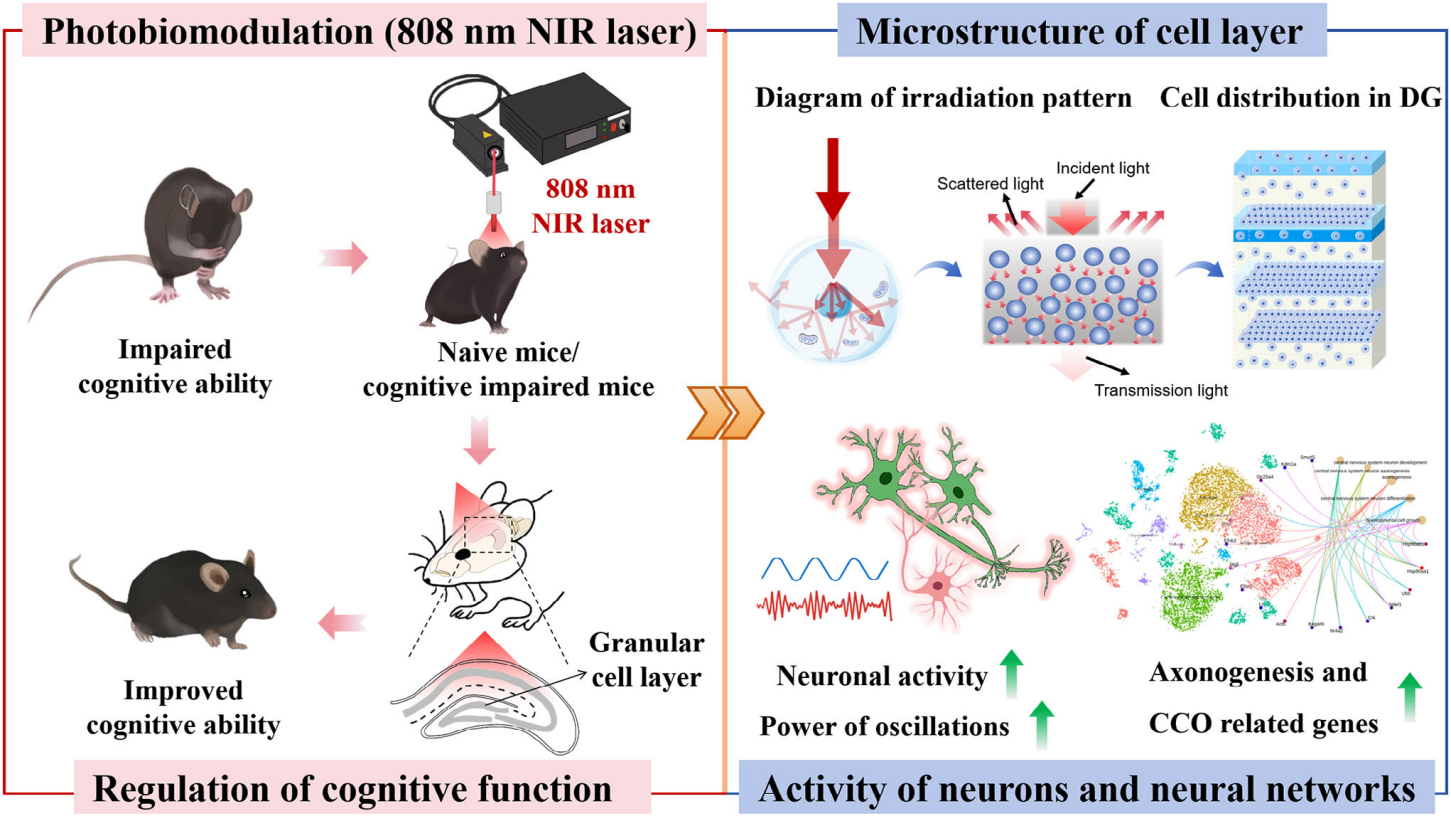
Schematic diagram of the phenotype, neuronal, and synapse levels of mice exposed to 808 nm NIR laser irradiation. This study developed a transcranial photo‐biomodulation approach using NIR laser on hippocampus of mice, revealing significant improvements in learning and memory ability of mice under normal and disease conditions. The improvement resulted from the high‐density cells induced local energy deposition and neuronal activation in the hippocampal dentate gyrus, suggesting a universal finding for the photo‐biomodulation of NIR laser.

Our study demonstrated that transcranial noninvasive irradiation of the hippocampus directly with 808 nm NIR laser enhanced the cognitive performance in naïve mice and attenuated cognitive impairment, induced by anesthesia and surgery or LPS injection, in the treated mice. Further analysis showed that this improvement in cognitive function was associated with enhanced neuronal activity in the high‐density cell layer of the hippocampal DG region. Specifically, single‐cell sequencing results showed that 808 nm NIR laser irradiation significantly activated axonogenesis and cytochrome C oxidase‐related genes in DG, which may be a key factor in the improvement of cognitive function by NIR laser treatment (**Figure** **8**).

The DG region is a subregion of the mammalian brain hippocampus that plays a key role in cognitive processes, including learning, memory formation, and pattern separation.^[^ ^23^ ^]^ Cognitively, DG is primarily responsible for the initial encoding and integration of memory.^[^ ^23^, ^24^ ^]^ The dorsal DG is preferentially involved in spatial learning and memory tasks, while the ventral hippocampus is more closely associated with emotional processing and regulation.^[^ ^25^ ^]^ Previous studies showed that selective regulation of dorsal DG selectively enhanced cognitive function without interfering with emotional processes, making it a promising intervention target for enhancing cognition.^[^ ^26^ ^]^ Hence, the NIR laser irradiation may provide a unique opportunity to treat cognitive impairment‐related brain functional disorders.^[^ ^4^ ^]^

Neural oscillations arise from synchronous interactions between neuronal groups and are fundamental to higher cognitive functions such as reasoning, decision making, control, and execution.^[^ ^27^ ^]^ Neural oscillations in different frequency bands are closely related to specific cognitive functions. High‐frequency oscillations (gamma bands) are generally associated with the performance of cognitive tasks and the integration of information processing, while low‐frequency oscillations (such as theta and alpha bands) are more associated with underlying cognitive processes such as emotion regulation and confident formation.^[^ ^28^ ^]^ Our results showed that among multiple irradiation intensities, only 6 mW cm^−2^ of NIR irradiation intensity significantly improved the cognitive ability of naïve mice, and the underlying mechanism is closely related to changes in the frequency or amplitude of multiband neural oscillations in the hippocampal DG region. Noticeably, this enhancement of energy effect does not only occur in a single band but is also the result of the common modulation of multiple bands, especially low‐frequency oscillation theta and alpha. Besides, high‐frequency oscillations of the field potential were likely to be modulated by low‐frequency oscillation phases. For example, theta oscillations were shown to coupling gamma oscillations, known as PAC, to enhance working memory by modulating synaptic plasticity, which has been well documented in both human and animal studies.^[^ ^15^, ^29^ ^]^ Our results further demonstrated that PAC was significantly increased in mice irradiated with 6 mW cm^−2^ of NIR laser. Previous studies showed that high gamma oscillations were closely related to retrospective spatial coding patterns, while low gamma oscillations were mainly responsible for predictive spatial coding patterns.^[^ ^30^ ^]^ Hence, the remarkably increased gamma oscillations induced by NIR may be mainly responsible for improved memory recall process in our study.

The effect of NIR stimulation on neural oscillations may depend on several factors, including the stimulus parameters, the target brain region, and the studied specific cognitive process.^[^ ^31^ ^]^ Our results showed that there was no significant change in the neuronal activity of the hippocampal CA1 region on the light path by NIR irradiation, and the activated neurons in the DG region was not cell specific, suggesting that NIR laser may not enhance the cognitive function by changing the biological mechanism of a certain class of neurons, but by enhancing the energy storage of NIR through the physical structure of special brain regions with specifically increasing overall neuronal activity in this brain region. From a physical point of view, when light penetrates into biological tissues, multioptical phenomena such as scattering, absorption, transmission, and refraction occur. Interestingly, the cell distribution in the hippocampus of mice means that the cell density in three clear structural bands is significantly higher than that of neighboring tissues. From the perspective of tissue refractive index and light scattering, tissues with dense cells have a lower tissue refractive index, and dense nuclei generate higher local light scattering to NIR, so NIR deposits higher light energy in the dense cells’ zone, which may be the reason why NIR laser activates neurons in the DG region.

Furthermore, our work showed that the energy deposition of 808 nm NIR laser in brain tissue can be regulated by microstructures created by different cell densities, which indicates that microstructures in biological tissues can induce changes in the optical path of NIR laser. Inspiringly, the energy accumulation of NIR laser in biological tissues can be manipulated by artificially adjusting or introducing optical structures, which may add new way of PBM. We can use optical materials with special microstructure to apply into the target brain regions to achieve a high degree of local light energy accumulation, and then specifically change neuronal activities to achieve artificial regulation of neural networks.

The observed increase in dendritic spine density and plasticity in DG neurons following NIR laser treatment suggests a structural basis for the enhanced learning and memory capabilities documented in both naïve mice and models of cognitive dysfunction.^[^ ^32^ ^]^ By promoting the growth and modification of dendritic spines, NIR laser irradiation may facilitate the strengthening of existing synapses and the formation of new ones, thereby enhancing the neural circuitry that supports cognitive processes.^[^ ^4^ ^]^ The molecular mechanisms underlying these structural changes likely involve the upregulation of genes related to axonogenesis and the modulation of CCO, as indicated by our single‐cell RNA sequencing data. The activation of these genes, along with the subsequent increase in CCO activity, enhanced mitochondrial function and energy metabolism,^[^ ^33^ ^]^ both of which are crucial for supporting the metabolic demands of synaptic plasticity. The observed enhancement of dendritic spine plasticity following NIR laser treatment opens promising new avenues for therapeutic interventions in neurocognitive disorders. Future research should explore whether these structural changes are accompanied by functional improvements in synaptic transmission and network activity.

C‐Fos is a common marker reflecting the neuronal activity. The expression may vary dynamically and be affected by various factors. Previous studies have found that the decrease of c‐Fos expression in cognitive dysfunction was caused by surgical anesthesia or inflammation.^[^ ^22^, ^34^ ^]^ In our study, we also affirmed that c‐Fos expression in DG was decreased in both POCD and LPS‐induced cognitive dysfunction model mice. Our electrophysiological data further demonstrate that the decreased neuronal activity in the DG might be a critical reason for cognitive dysfunction. Besides, numerous literatures have proved that neuroinflammation is also an important cause of cognitive dysfunction.^[^ ^35^ ^]^ More importantly, the cognitive impairment induced by neuroinflammation was due to the decrease of c‐Fos expression.^[^ ^36^ ^]^ Hence, we consider that NIR laser might improve the cognitive function of POCD and LPS‐induced cognitive dysfunction model mice by reducing neuroinflammation and restoring neuronal activity.

Intraperitoneal injection of LPS may affect other brain regions in mice except the hippocampus. Whether other brain regions can be activated by NIR to improve mouse cognition is what we need to investigate in future studies. Notably, previous studies reported that the mice with 0.5 mg kg^−1^ LPS injection did not affect certain cognitive behaviors, such as the Y maze.^[^ ^37^ ^]^ In our study, 0.5 mg kg^−1^ LPS injection caused mice poor cognitive and behavioral performance. This may be due to the difference in the frequency of LPS injected and the time points of the behavioral paradigm. Besides, the genetic type of mice used may also be a major reason.

This special energy‐storing effect of NIR is applicable to multiple brain regions. We only studied the hippocampus DG, which is closely related to cognitive function, and other brain regions, such as medial prefrontal cortex (mPFC), are also promising intervention targets for cognition. There are various anatomical connections between the hippocampus and mPFC, and neural oscillations can be regarded as the bridge of communication between hippocampus and mPFC. However, whether NIR laser can regulate the neuronal activity of mPFC by interfering with neural oscillations of DG region remains unclear, and further studies are needed. Moreover, our experiment was only verified in animal models, and because the complicated structure of human brain is different from that of mice, intervention parameters and intervention modes of NIR light need to be studied further clinically for cognitive disorders such like Alzheimer's disease, POCD, and depression. Despite their differences in clinical presentation and disease pathophysiology, these diseases share some key pathophysiological features, such as impaired neuronal communication, neuroinflammation, oxidative stress, and altered levels of neurotrophic factors.^[^ ^38^ ^]^ The potential mechanisms of the NIR laser likely ameliorate these shared pathophysiological processes, and patients with various neurological diseases may be benefited from this noninvasive therapy per se.

Our study has some limitations. First, we found that c‐Fos expression in DG was decreased in both POCD and LPS‐induced cognitive dysfunction models. Previous study reported that the recovery of c‐Fos expression after surgery tended to be normalized on day 7.^[^ ^39^ ^]^ However, our study found that the reduce of c‐Fos expression persisted on day 7, which is possibly due to the use of adult mice rather than aged mice in our experiments. C‐Fos expression may vary dynamically, and its expression level is affected by various factors, including the type and timing of surgery, the mode of intervention, and the duration of intervention, among others. Its cause and effect association in our study is unknown. However, electrophysiological data that corroborate other changes including c‐Fos provide assurance in supporting our conclusions. Second, in our study, the NIR fiber was positioned directly on the upper part of the hippocampus, meaning that the NIR irradiation covered the entire hippocampal region, rather than being limited to the DG region. While our results demonstrated that NIR laser primarily enhanced neuronal activity in the hippocampal DG region, this does not rule out the possibility that the NIR laser may also affect the CA1 and CA3 regions. Therefore, cognitive enhancement by NIR may involve the entire hippocampus rather than the DG region alone, and this warrants further study.

In summary, our study demonstrated that transcranial, noninvasive irradiation of the hippocampus with a continuous 808 nm NIR laser (5 min per day for 5 days) enhanced cognitive performance in both naïve and cognitively impaired mice. An intriguing aspect of our findings is that NIR specifically enhanced neuronal activity in the hippocampal DG region. The NIR laser induced localized energy deposition in high‐density cellular tissues, providing an important theoretical foundation for PBM with NIR laser. PBM facilitated strong gene transcriptome, molecular changes, and axonogenesis toward cognitive improvements. However, the efficacy of this approach in improving cognition in other neurological disease models needs further validation before clinical trial studies can be initiated.

## 4 Experimental Section

### Animals

Adult male C57BL/6J mice (8–12 weeks old) were purchased from Beijing SiPeiFu Biotechnology Co., Ltd., Beijing, China. Adult male VGluT2‐ires‐cre mice (Jackson Laboratory, USA, JAX#016963) were kindly provided by Dr. Min‐Min Luo from the National Institute of Biological Sciences, China. All animals were group‐housed in a temperature‐ and humidity‐controlled (23 ± 2 °C, 50–60%) room under a 12 h dark–light cycle, with free access to water and food. All procedures were performed in compliance with the International Association for the Study of Pain and were approved by the Institutional Animal Care and Use Committee of The First Affiliated Hospital of Zhengzhou University (2023‐KY‐0135‐001). All efforts were made to minimize suffering and number of animals used.

### Behavioral Test

A well‐trained researcher conducted the behavioral tests, including the Barnes maze and Y maze tests in a blinded manner.

In the Barnes maze test, a circular platform with 20 equidistant holes, including one leading to a dark refuge, was used to evaluate the spatial learning and memory in mice.^[^ ^22^, ^40^ ^]^ Mice were preadapted to the target hole environment for 4 min the day before the training day. During the 5 day training phase (once a day, 4 min each time), a fiber optic was attached to the mouse's head to monitor behavior, and a 200 W light source was positioned above the platform to motivate the mice to locate the target hole without obstructing the fiber optic. Short‐ and long‐term spatial memory tests were performed on days 1 and 7 after training, respectively. No experiments were performed from day 2 to day 6 after training. The SuperMaze system was utilized to record the latency to find the target hole and the mice's movement trajectories.

The Y maze test was performed as described previously.^[^ ^41^ ^]^ Briefly, the SuperMaze system was used to acquire images and analyze spontaneous alternation, in which a mouse chose to enter three arms consecutively (ABC, CAB, or BCA, but not BCB, CBC, or CAC). The ratio of spontaneous alternation was calculated as follows: (number of spontaneous alternations)/(total number of arm entries − 2) ×100.

### Anesthesia and Surgery

Right carotid artery was exposed 1–1.2 cm without damage to the vagus nerve under anesthesia with 1.5–2% isoflurane as well documented.^[^ ^42^ ^]^ The procedure was performed under aseptic conditions and lasted about 10 min. After 2 h of anesthesia, mice were freed from isoflurane and awakened.

### LPS Treatment

LPS (Sigma–Aldrich, St. Louis, MO; O111:B4, 0.5 mg kg^−1^) was diluted in normal saline and administered intraperitoneally to induce cognitive deficits in mice.^[^ ^12a^ ^]^ The control group received the same volume of normal saline.

### In Vivo Electrophysiological Recording

For in vivo electrophysiological recording, a 4 × 2 electrode array that consisted of eight microwires (25 µm in diameter, Kedou, Suzhou, China) was implanted into the DG. Two screws were fixed on the skull and attached to stainless steel wires as ground. After surgery, the mice were kept separately and were allowed to recover for 5–7 days. All mice were habituated to the recording headstage for 10 min per day for 2–3 days in the test environment. Mice were placed in a square box wrapped with a copper mesh (freely moving test). In vivo signals were recorded by an Apollo II 32ch digital signal processor (Bio‐Signal Technologies, Nanjing, China) and amplified, filtered at a 300–5000 Hz bandwidth and stored by Apollo II 32ch acquisition software.

For LFP analysis, the signals were transmitted into the Apollo II 32ch digital signal processor and digitized at a sampling rate of 40 kHz. Theta (4–8 Hz), alpha (8–13 Hz), beta (13–30 Hz), low‐gamma (30–50 Hz), medial‐gamma (50–80 Hz), and high‐gamma (80–120 Hz) rhythms were filtered, and the mean power of each specific rhythm was verified by averaging across the frequency bands. LFPs were filtered between 1 and 120 Hz and sampled at 2 kHz. The PAC between gamma amplitude and theta phase was measured by using the python. The bandwidth for filtered phase frequency was 4 Hz. The bandwidth for filtered amplitude frequency was 10 Hz.^[^ ^15^, ^43^ ^]^

For spikes, well‐isolated units were classified using an unsupervised clustering algorithm. Spiking signals were extracted from the field potential signals after high‐pass filtering (≥300 Hz) sampled at 30 kHz based on a *κ*‐means method. The spikes were sorted offline based on three principal components (half‐spike width, half‐valley width, and mean firing rate).^[^ ^44^ ^]^

All data were analyzed with an Offline Sorter (version 2.8.5; Plexon, USA) and Neuroexplorer (Nex Technologies, USA).

### Fiber Photometry

Calcium‐dependent signals were recorded using a fiber photometry system (INPER, Hangzhou, China), with the 470 nm light emitting diode (LED) power of 45 mW and the 410 nm LED power of 30 mW. Immunofluorescence was performed at the injection site after behavioral testing, and mice with no virus expression were excluded from the data analysis. For fiber photometry with immediate NIR laser intervention, mice were placed in a square plastic box (50 × 50 × 50 cm^3^) and allowed free movement. Calcium‐dependent signals were recorded at the instant of NIR laser on and off, and two trials with repeated stimulation were performed for each mouse. After the end of the first trail, the second trail was started after waiting at least 10 min.

For fiber photometry during behavioral testing, mice were placed on the disk tested by Barnes maze and allowed free movement. Calcium‐dependent signals were recorded throughout the Barnes and the Y maze tests. Data analysis was performed using InperPlot software (Inper Ltd., Hangzhou, China). For Barens maze and immediate NIR laser intervention, values of Ca^2+^ transients change (Δ*F*/*F*) from −4 s (or −8 s) to 8 s (0 s, stimuli onset) were derived by calculating (*F* − *F*0)/*F*0 for each trial. The *F*_0_ value was selected as dynamic (30 Hz sampling rate, *τ*_0_ = 0.2 s, *τ*_1_ = 0.75 s, and τ_2_ = 3 s) to better exclude the possible baseline drift caused by high‐power laser and the interference caused by high‐frequency noise. Values of peak and area under cure (AUC) were normalized with InperPlot software. Subjects with off‐target fiber ends’ location were excluded from analysis. In addition, Y maze calcium signal analysis was referred to previous paradigms,^[^ ^45^ ^]^ and only Δ*F*/*F* values exceeding three standard deviations were classified as events.

### Immunohistochemistry

Immunohistochemistry was performed according to the earlier study.^[^ ^44^ ^]^ The detailed procedures are presented in the Supplementary Methods (Supporting Information).

### Golgi Staining

Golgi staining was performed according to the manual and earlier study.^[^ ^12b^ ^]^ The detailed procedures are presented in the Supplementary Methods (Supporting Information).

### In Vitro Optical Validation Test

For the scattering effect of NIR laser in the hippocampus of mouse, 5 µm monodispersed PS nanospheres (9003‐53‐6, NanJing Nanorainbow Biotechnology Co., Ltd.) were used for in vitro hippocampal simulation. The density of PS nanospheres was 1.0 g cm^−3^, and the solid content was 10%. The light absorption of particles solution was measured by a UV–vis spectrophotometer (Japan JASCO, V‐550). The absorbance, transmittance, and reflectance spectra of the suspension of PS microspheres were measured with a fiber optical spectrometer (Oceanoptics HR2000CG‐UV‐NIR) at wavelengths ranging from 550 to 900 nm.^[^ ^46^ ^]^

All the simulations were performed in an FDTD method. A plane wave of 808 nm was used as the light source throughout experiments. The boundary conditions were periodic boundary conditions (PBC) in the *X–Y* direction and perfectly matched layer (PML) in the *Z*‐direction. The mesh size was tuned to obtain a good tradeoff between the computer memory requirements and the simulation time, while ensuring convergence of the results. A convergence test was carefully performed.^[^ ^47^ ^]^

### Single‐Cell RNA Sequencing

Hippocampal DG regions were harvested after 5 days of 808 nm NIR laser irradiation for single‐cell RNA sequencing (*n* = 4 per group). Single‐cell RNA sequencing was performed according to the protocols of 10× Genomics, and the detailed process of the data analysis is presented in the Supplementary Methods (Supporting Information).

### Statistical Analysis

Statistical tests were analyzed with GraphPad Prism (GraphPad, CA, USA, version 9.0). A normality test was applied for all data before analysis. All data were presented as the mean ± s.e.m. For behavioral and immunofluorescence data, two‐tailed paired Student's *t*‐test, two‐way repeated measures analysis of variance (ANOVA) with Bonferroni's multiple comparisons test, or one‐way repeated measures ANOVA with Bonferroni's multiple comparisons test was used wherever appropriate. For in vivo electrophysiological data, Kruskal–Wallis ANOVA with a posthoc Dunn's test, one‐way repeated measures ANOVA with Bonferroni's multiple comparisons test, or two‐way repeated measures ANOVA with Bonferroni's multiple comparisons test was used as appropriate. For fiber photometry data, the two‐tailed paired Student's *t*‐test was used. Differences were considered significant at *p* < 0.05.

## Conflict of Interest

The authors declare no conflict of interest.

## Author Contributions

W.‐T.P. and H.‐W.G. contributed equally to this work. P.‐M.L. and W.‐T.P. contributed to conceptualization; W.‐T.P., H.‐W.G., and Y.‐B.Z. contributed to methodology; W.‐T.P. and P.‐M.L. contributed to Visualization; J.‐J.Y. and P.‐M.L. contributed to funding acquisition; J.‐J.Y. contributed to project administration; J.‐J.Y. and D.Q.M. contributed to supervision; W.‐T.P., P.‐M.L., H.‐W.G. contributed to writing the original draft; and J.‐J.Y., D.Q.M., S.M., and P.‐M.L. contributed to writing the review and editing.

## Supporting information



Supporting Information

## Data Availability

The data that support the findings of this study are available in the Supporting Information of this article.
