# Supplementary material for: Photo‐Biomodulation of the Hippocampus Using Near‐Infrared Laser to Enhance Cognitive Function in Mice
Source: Adv Sci (Weinh). 2025 Apr 7;12(22):2417380. doi: 10.1002/advs.202417380 (PMC12165041; doi:10.1002/advs.202417380)
Supplement: Supplementary file 1 — Supporting Information [file ADVS-12-2417380-s001.docx]

**Supplemental** **information**

**Photobiomodulation of the hippocampus using near-infrared laser to enhance cognitive function in mice**

Wei-Tong Pan, Han-Wen Gu, Yan-Bo Zhou, Sophie Ma, Daqing Ma, Pan-Miao Liu, Jian-Jun Yang

**List of Supplementary Material**

1. **Supplementary Methods**
2. **Supplementary Figures**

Figure S1: Characterization of 808 nm NIR laser irradiation.

Figure S2: 808 nm NIR laser effect in enhancing the spatial learning and memory for naïve mice lasted for two days.

Figure S3: Representative histological images of cortex and hippocampus after laser irradiation.

Figure S4: NIR instrument energy index and laser transmittance test.

Figure S5: Analysis of local field potentials in the DG.

Figure S6: Representative traces of high-pass-filtered LFPs of control and NIR-irradiation groups.

Figure S7: Analysis of local field potentials in the CA1.

Figure S8: Calculation of the change of spikes in DG.

Figure S9: Calculation of the change of spikes in CA1.

Figure S10: Population Ca2+ activity in DG neurons during Y-maze (8 min).

Figure S11: The activation effect of 808 nm NIR laser on excitatory neurons in VGluT2-ires-cre mice excluding the confounding factors.

Figure S12: Calculation of PS microspheres concentration according to the hippocampus slices.

Figure S13: SEM (scanning electron microscope) images and real shots of the prepared PS microsphere solution.

Figure S14: The optical characterization of PS microspheres ranging from 550 nm to 900 nm of visible-near-infrared waveband.

Figure S15: Expression of c-Fos in the hippocampus of mice after 1064 nm NIR laser irradiation.

Fig. S16: Single-cell RNA sequencing results of cells in DG (1).

Fig. S17: Single-cell RNA sequencing results of cells in DG (2).

Fig. S18: Single-cell RNA sequencing results of cells in DG (3).

Fig. S19: 808 nm NIR laser rescued cognitive dysfunction and improved the energy of multiple frequency and theta-gamma coupling of DG of POCD model mice.

Fig. S20: 808 nm NIR laser rescued cognitive dysfunction of LPS-induced cognitive dysfunction model mice.

Fig. S21 Changes in TNF-α in the DG of cognitive dysfunction model mice.

1. **Supplementary Methods**

***Immunohistochemistry***

Briefly, mice were transcardially perfused with 0.9% saline and 4% paraformaldehyde (PFA) under isoflurane anaesthesia. After perfusion, brains were removed, postfixed in the same fixative overnight at 4 °C, and cryoprotected in 30% sucrose phosphate-buffered saline for at least 72 h. Brain sections of 30 μm were prepared using a freezing microtome (Leica, Germany, CM1950). The sections were randomly selected and blocked with 5% goat serum in 0.3% Triton X-100 for 1 hour at room temperature. Then, they were incubated with primary antibody rabbit anti-c-Fos (1:400, Cell Signaling Technology, USA, #2250) overnight at 4 °C. After being washed with PBS, sections were incubated with secondary antibody goat anti-rabbit Cy3-conjugated secondary antibody (1:200, Jackson ImmunoResearch, USA, 111 –165-003) for 2 h at room temperature. The stained sections were mounted onto slides with DAPI Fluoromount-G mounting medium (SouthernBiotech, USA, 0100–20) and examined with an Olympus BX53 fluorescence microscope (Olympus Optical, Tokyo, Japan).

***Golgi staining***

In brief, mice were irradiated with 808 nm NIR laser (control mice were not irradiated) and brain tissues were rapidly removed after isoflurane anesthesia, and processed using the FD Rapid GolgiStain^TM^ Kit (FD Neurotechnologies, Inc., Columbia, MD, USA) . After rinsing with normal saline, the brain was rinsed in a fresh mixture of solution A (3.0 ml) and solution B (3.0 ml) for two weeks in the dark at RT. The mixture solution was replaced after the initial 24 h of immersion. Following this step, the tissue was transferred to solution C and stored in the dark for one week, with a replacement of the solution after the first 24 h. Subsequently, slices with a thickness of 110 μm were sectioned using a freezing microtome (Leica, Germany, CM1950) and dried in the dark. Next, the sections were washed twice for 4 min each with milli-Q water and then placed in a mixture (50 ml) consisting of solution D, solution E, and milli-Q water, with a volume ratio of 1:1:2. This mixture was allowed to interact with the sections for 10 min. Following this, the sections were rinsed twice for 4 min each with milli-Q water, dehydrated using graded ethanol, cleared with xylene, and finally covered with a slide using permount. Dendritic morphology of neurons in the DG were captured with a confocal microscope at 20× and 100× objectives (Olympus FluoView FV1000, Tokyo, Japan). Their morphology and dendritic spines were analyzed with Neuron J plugin, Sholl analyses, and Cell Counter plugin with Image J software.

***Hematoxylin-eosin staining***

The brain blocks were fixed with 4% PFA, embedded in paraffin and then sectioned with a microtome (RM2016, Shanghai Leica Instrument) to sections of 4 mm thickness. They were then stained with hematoxylin-eosin staining and the pathological changes of cortex and hippocampus (CA1 and DG) were assessed under a light microscope (200×).

***Stereotaxic surgery, virus injection and fiber optic cannula implantation***

Mice were deeply anaesthetized with isoflurane (2–3%) through a nose cone, and then placed in a stereotactic frame (RWD Life Science Co.,LTD, Shenzhen, China). A heating pad was used to maintain the body temperature of mice at 37 ± 0.2◦C. Scalp hair was removed using hair removal cream. Erythromycin eye cream was applied to the eyes to keep them moist. A midline incision was made to expose the skull.

For virus injection, a small craniotomy was performed at the target site using a hand-held cranial drill (RWD Life Science Co.,LTD) under anaesthesia. All viruses were injected into the DG [anteroposterior (AP) from bregma: -1.9 mm; mediolateral (ML) from midline: ±1.0 mm; dorsoventral (DV) from dura: -2.0 mm] *via* a 5 μL microsyringe (RWD Life Science Co, Shenzhen, China) at a volume of 200 nL per side at a rate of 25 nL/min. After injection, the needle was kept in the site for 8 min. After the needle was removed, the 0.2 mm diameter fiber optic cannulae (NA=0.37, INPER, China) were implanted in the same position until the tip was 0.1-0.2 mm above the virus injection target and fixed in place with denture base resins. Postoperative recovery and viral expression required 6-8 weeks.

For mice exposed to NIR laser alone, an optic fiber cannula was placed above on the skull surface above the DG and secured with denture base resins to ensure that it was stable and did not compress the skull. The cannula placement side was randomly selected. After surgery, mice were placed under a homeothermic heating pad until awakening and further monitored daily. Postoperative recovery was allowed up to 3 days after surgery.

***Apparatus and treatment***

The laser apparatus used was a 808 nm single-mode fiber coupled laser (MW-GX-808/5000mW, Changchun Laser Optoelectronics Technology Co., Ltd.). A mono-fiber optic was selected for the laser wire (Fiber Interface: FC/PC, 5m, NA=0.37) according to the machine's own P-I [POWER(W)-IOA(A)] curve. In all experiments, the NIR laser irradiation intensity or power received by mice was based on the output laser at the end of the fiber optic.

The intensity of NIR laser irradiation was measured with an Infrared Radiometer (LH-131, Shenzhen Lianhuicheng Technology Co., LTD, Shenzhen, China). The power of NIR laser irradiation was measured with a laser power meter (LP10, SANWA ELECTRIC INSTRUMENT CO, LTD.)

The temperature of mouse body surface and skull surface was measured with a hand-held thermal camera (H13, Hangzhou Hikvision Digital Technology Co., Ltd.). A contact thermocouple thermometer was selected for temperature measurement in the mouse brain (YET-610, OMEGA Engineering).

In the *in vivo* electrophysiological experiments, mice were freely moved in an opaque chamber (50×50×50cm), and the NIR laser irradiation was used simultaneously during the electrophysiological measurements. In the gradient laser illumination experiment, each mouse was exposed to different laser irradiation intensities from low to high power for 2 minutes, and then the laser was turned off for 10 minutes before the next irradiation test. In the naïve mice behavior training, the irradiance of the laser at the end of the wire was 0, 0.6, 6, and 60 mW/cm^2^. In the disease model behavioral training, the irradiance of the laser at the end of the wire was 6 mW/cm^2^. The NIR laser irradiation was used simultaneously during training.

***Western blot analysis***

Utilizing a previously established methodology with certain modifications[1], we conducted Western blotting (WB) to assess the protein expression levels of tumor necrosis factor-alpha (TNF-α) within the bilateral dorsal hippocampal DG region. Following administering anesthesia via 5% isoflurane, the hippocampal tissue was promptly excised and placed on ice to preserve its integrity. Briefly, hippocampal DG tissues were ground in RIPA buffer (CWBIO Co., Ltd., Cat Number: 01408/28421, Taizhou, Jiangsu, China) containing protease and phosphatase inhibitor cocktail (CWBIO Co., Ltd., Cat Number: CW2200S, Taizhou, Jiangsu, China). The lysates were centrifuged at 12000 g at 4℃ for 5 min, and the supernatants’ protein concentrations were measured by a bicinchoninic acid assay (BCA) (Cat Number: GK10009, Glpbio Technology Inc., Montclair, CA, USA). The protein samples were heated for 5 min and then frozen at -20℃ until use. Then, 20 μg of protein was loaded, separated by 10% sodium dodecyl sulfate polyacrylamide gel electrophoresis (SDS-PAGE) (Bio-Rad, Hercules, CA, USA) and transferred onto polyvinylidene fluoride membranes (PVDF) membranes (0.45 μm pore size, Sigma-Aldrich Co., Ltd., St Louis, MO, USA). The membranes were blocked in 5% nonfat dry milk in Tris-buffered saline-Tween 20 (TBST) for 1 h at room temperature, incubated overnight at 4℃, and treated with primary antibodies: rabbit anti-TNF-α (1:1000, Cat Number: A11534, ABclonal Co., Ltd., Wuhan, Hubei, China), mouse anti-β-actin (1:5000, Cat Number: 66009-1-LG, Proteintech Group Co., Ltd., Wuhan, Hubei, China) and anti-β-Tubulin (1:5000, Cat Number: A12289, ABclonal Co., Ltd., Wuhan, Hubei, China. Then, the membranes were rinsed with TBST thrice for 5 min each and subsequently incubated with goat anti-rabbit (1:5000, Cat Number: 0295G, Bioss Co., Ltd., Beijing, China) or goat ant-mouse (1:5000, Cat Number: 0296G, Bioss Co., Ltd., Beijing, China) secondary antibodies for 2 h at room temperature. The target proteins were visualized with an ECL Plus detection kit (Thermo Fisher Scientific, Rockford, IL, USA), and densitometry quantification of the WB signal was conducted using ImageJ software.

***Single-cell RNA sequencing and analysis***

1. Tissue dissociation and cell purification

Tissues were transported in sterile culture dishes with 10 ml 1x Dulbecco's Phosphate-Buffered Saline (DPBS; Thermo Fisher, Cat. no. 14190144) on ice to remove the residual tissue storage solution, then minced on ice. We used dissociation enzyme 0.25% Trypsin (Thermo Fisher, Cat. no. 25200-072) and 10 ug/mL IDNase I (Sigma, Cat. no. 11284932001) dissolved in PBS with 5% Fetal Bovine Serum (FBS; Thermo Fisher, Cat. no. SV30087.02) to digest the tissues. Hippocampus tissues were dissociated at 37 C with a shaking speed of 50 r.p.m for about 40 min. We repeatedly collected the dissociated cells at interval of 20 min to increase cell yield and viability. Cell suspensions were filtered using a 40 μm nylon cell strainer and red blood cells were removed by 1X Red Blood Cell Lysis Solution (Thermo Fisher, Cat. no. 00-4333-57) . Dissociated cells were washed with 1x DPBS containing 2% FBS. Cells were stained with 0.4% Trypan blue (Thermo Fisher, Cat. no. 14190144) to check the viability on Countess@ I Automated Cell Counter (Thermo Fisher).

1. Single nucleus isolation and sequencing procedures

Nuclei were isolated using a Shbio Nuclei Isolation Kit (SHBIO, #52009-10, China). Briefly, the frozen tissue samples were quickly added to the lysis buffer and the tissue was ground to liquid state using a tissue homogenizer. The tissue lysate was passed through a 40 μm cell sieve to remove impurities and transferred to a new 2mL EP tube and centrifuge at 500 g at 4 ° C for 5 min to obtain the precipitation. Next, PB1, PB2, PB3 solutions were added to the precipitation and the nuclei were located at the junction of PB2 and PB3 solutions. Finally, the nuclei were resuspended by blowing into 50 μL NB solution. Nuclei were counted with a cell counter (Thermo Fisher, America). Using a Chromium Single Cell 3′ Library and Gel Bead Kit v3 (10X Genomics, America), nuclei were immediately loaded onto a Chromium Single Cell Processor(10X Genomics, America) for barcoding of RNA from single nuclei. Sequencing libraries were constructed according to the manufacturer’s instructions (10× Genomics, America) and then sequenced in a NovaSeq 6000 sequencing system (Illumina, America).

1. 10× library preparation and sequencing

Beads with unique molecular identifier (UMI) and cell barcodes were loaded close to saturation, so that each cell was paired with a bead in a Gel Beads-in-emulsion (GEM). After exposure to cell lysis buffer, polyadenylated RNA molecules hybridized to the beads. Beads were retrieved into a single tube for reverse transcription. On cDNA synthesis, each cDNA molecule was tagged on the 5’ end (that is, the 3’ end of a messenger RNA transcript) with UMI and cell label indicating its cell of origin. Briefly, 10× beads that were then subject to second-strand cDNA synthesis, adaptor ligation, and universal amplification. Sequencing libraries were prepared using randomly interrupted whole-transcriptome amplification products to enrich the 3’ end of the transcripts linked with the cell barcode and UMI. All the remaining procedures including the library construction were performed according to the standard manufacturer’s protocol (CG000206 Rev D). Sequencing libraries were quantified using a High Sensitivity DNA Chip (Agilent) on a Bioanalyzer 2100 and the Qubit High Sensitivity DNA Assay (Thermo Fisher Scientific). The libraries were sequenced on NovaSeq6000 (Illumina) using 2x150 chemistry.

1. Single cell RNA-seq data processing

Reads were processed using the Cell-Ranger v5.0.0 pipeline with default and recommended parameters. FASTQs generated from Illumina sequencing output were aligned to the mouse genome, version GRCm38, using the STAR algorithm.[2] Next, Gene-Barcode matrices were generated for each individual sample by counting UMIs and filtering non-cell associated barcodes. Finally, we generate a gene-barcode matrix containing the barcoded cells and gene expression counts. This output was then imported into the Seurat (v3.0.2) R toolkit for quality control and downstream analysis of our single cell RNAseq data.[3] All functions were run with default parameters, unless specified otherwise. We excluded cells with fewer than 200 or more than 6000 detected genes (where each gene had to have at least one UMI aligned in at least three cells). The expression of mitochondria genes was calculated using PercentageFeatureSet function of the seurat package.[3] To remove low activity cells, cells with more than 10 percent expression of mitochondria genes were excluded. The normalized data (NormalizeData function in Seurat package) was performed for extracting a subset of variable genes. Variable genes were identified while controlling for the strong relationship between variability and average expression. Next, we integrated data from different samples after identifying ‘anchors’ between datasets using FindIntegrationAnchors and IntegrateData in the seurat package.[3-4] Then we performed principal component analysis (PCA) and reduced the data to the top 30 PCA components after scaled the data. We visualized the clusters on a 2D map produced with t-distributed stochastic neighbor embedding (t-SNE).[5]

1. Identification of cell types and subtypes by nonlinear dimensional reduction (t-SNE)

Cells were clustered using graph-based clustering of the PCA reduced data with the Louvain Method [6] after computing a shared nearest neighbor graph.[3] For sub-clustering, we applied the same procedure of scaled, dimensionality reduction, and clustering to the specific set of data (usually restricted to one type of cell). For each cluster, we used the Wilcoxon Rank-Sum Test to find significant deferentially expressed genes to compare the remaining clusters. SingleR [7] and known marker genes were used to identify the cell type.

1. Single-cell sequencing cell type annotation

The single-cell sequencing cell type annotation software SingleR, websites (CellMarker 2.0, panglaodb, proteinatlas) and marker gene combination were used to annotate the cell types. Ldb2, Cdh18, Camk2, Il1rapl2, Tshz2, Cpne4 and Htr2c are canonical markers for excitatory neurons. Adarb2, Trps1, Erbb4, Nxph1 and Npas3 are canonical markers for inhibitory neurons. Slc4a4, Trpm3 and Slc1a3 are canonical markers for astrocytes. Hexb is a canonical marker for microglia. SPlp1, Dpp10, Lhfpl3, Plp1 are canonical markers for oligodendrocyte. Galntl6 is present in both excitatory and inhibitory neurons, so its cluster as a marker can only be defined as excitatory and inhibitory neuron. Other clusters (Gm28928, Gm26883, Tshz2 and Mgat4c) are not defined.

**Reference**

[1] a) C. Yue, W. Luan, H. Gu, D. Qiu, X. Ding, P. Liu, X. Wang, K. Hashimoto, J. J. Yang, *J Psychiatr Res* **2023**, *166*, 61, <https://doi.org/10.1016/j.jpsychires.2023.09.003>; b) T. T. Zhu, H. Wang, H. W. Gu, L. S. Ju, X. M. Wu, W. T. Pan, M. M. Zhao, J. J. Yang, P. M. Liu, *J Nanobiotechnology* **2023**, *21* (1), 52, <https://doi.org/10.1186/s12951-023-01807-4>.

[2] D. T. Le, J. N. Durham, K. N. Smith, H. Wang, B. R. Bartlett, L. K. Aulakh, S. Lu, H. Kemberling, C. Wilt, B. S. Luber, F. Wong, N. S. Azad, A. A. Rucki, D. Laheru, R. Donehower, A. Zaheer, G. A. Fisher, T. S. Crocenzi, J. J. Lee, T. F. Greten, A. G. Duffy, K. K. Ciombor, A. D. Eyring, B. H. Lam, A. Joe, S. P. Kang, M. Holdhoff, L. Danilova, L. Cope, C. Meyer, S. Zhou, R. M. Goldberg, D. K. Armstrong, K. M. Bever, A. N. Fader, J. Taube, F. Housseau, D. Spetzler, N. Xiao, D. M. Pardoll, N. Papadopoulos, K. W. Kinzler, J. R. Eshleman, B. Vogelstein, R. A. Anders, L. A. Diaz, Jr., *Science* **2017**, *357* (6349), 409, <https://doi.org/10.1126/science.aan6733>.

[3] R. Satija, J. A. Farrell, D. Gennert, A. F. Schier, A. Regev, *Nat Biotechnol* **2015**, *33* (5), 495, <https://doi.org/10.1038/nbt.3192>.

[4] T. Stuart, A. Butler, P. Hoffman, C. Hafemeister, E. Papalexi, W. M. Mauck, 3rd, Y. Hao, M. Stoeckius, P. Smibert, R. Satija, *Cell* **2019**, *177* (7), 1888, <https://doi.org/10.1016/j.cell.2019.05.031>.

[5] Maaten L, H. G. V, *Journal of machine learning research* **2008**, *9*, 2579.

[6] V. D. Blondel., J.-L. Guillaume., R. Lambiotte., E. Lefebvre, *Journal of statistical mechanics: theory and experiment* **2008**, *10*, P10008, <https://doi.org/10.48550/arXiv.0803.0476>.

[7] D. Aran, A. P. Looney, L. Liu, E. Wu, V. Fong, A. Hsu, S. Chak, R. P. Naikawadi, P. J. Wolters, A. R. Abate, A. J. Butte, M. Bhattacharya, *Nat Immunol* **2019**, *20* (2), 163, <https://doi.org/10.1038/s41590-018-0276-y>.

1. **Supplementary Figures**


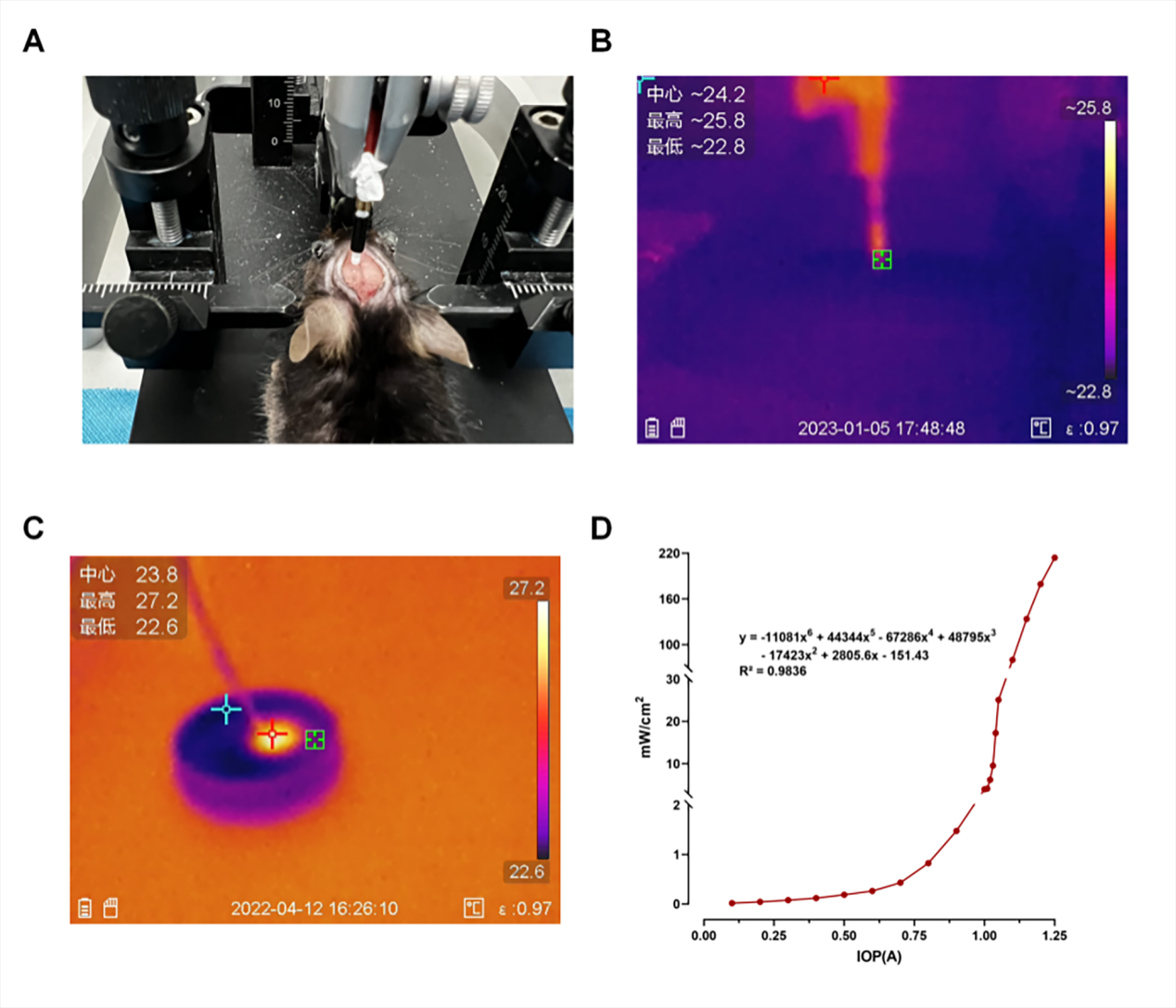


**Fig. S1. Characterization of 808 nm NIR laser irradiation. A** Top view of the mouse skull surface with fiber optic cannula fixation. **B-C** The results of temperature measurement in the air and water. **D** The laser machine's P-I [POWER(W)-IOA(A)] curve. The laser irradiance power (mW/cm^2^) was changed by adjusting the current knob (IOP) of the NIR laser.


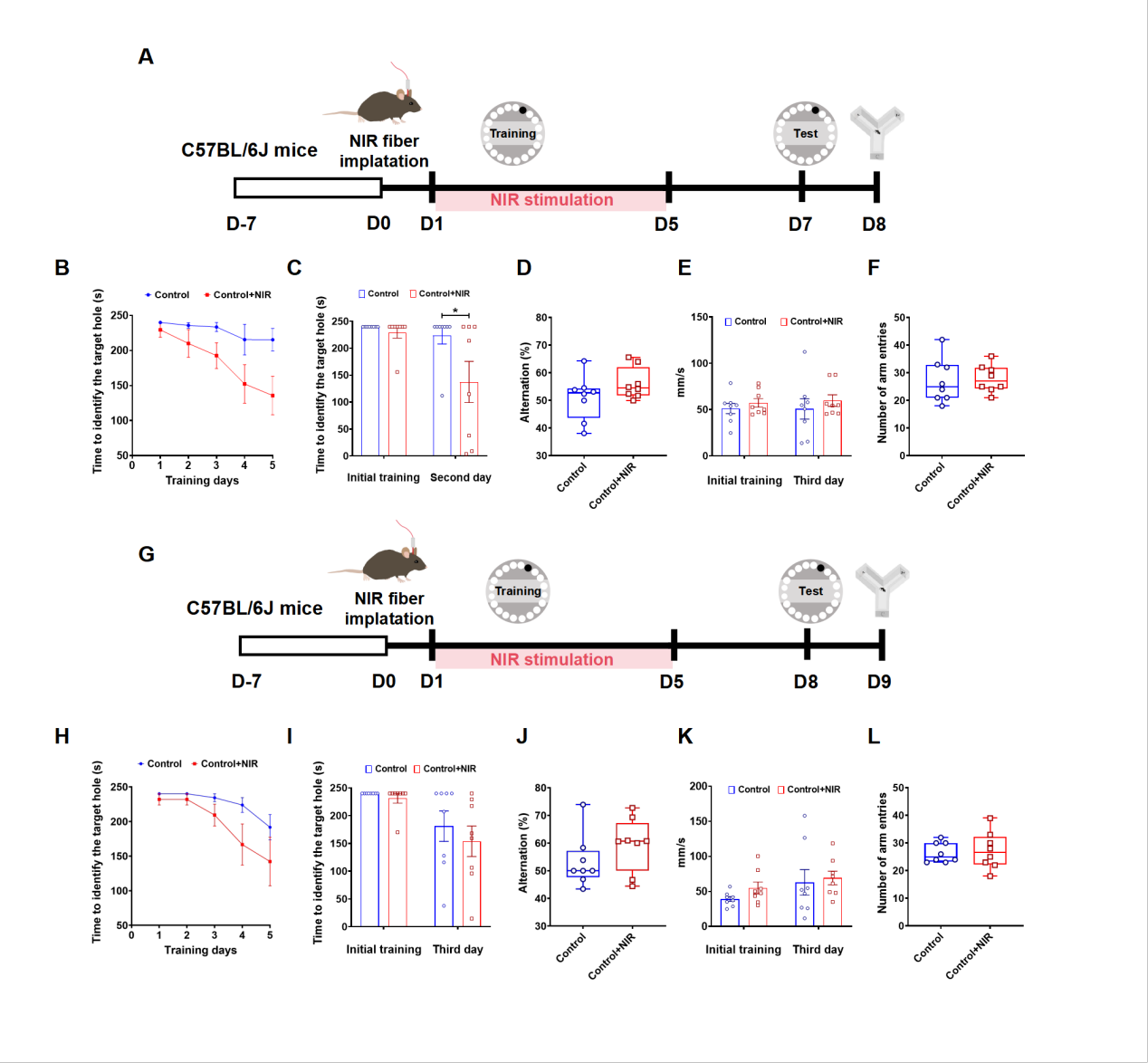


**Fig. S2. 808 nm NIR laser effect in enhancing the spatial learning and memory for naïve mice lasted for two days. A** Experimental schematic of 808 nm NIR laser irradiation and behavior tests for naïve mice (tested on the second day after training) **B,C** Identify latency of the training period (**B**), identify latency of the test (**C**) of mice of the Barnes maze test (Interaction: F_(1, 28)_=3.152, *P*=0.0867, Test day: F_(1, 28)_=6.365, *P*=0.0176, Group: F_(1, 28)_=5.134, *P*=0.0314) **D** The proportion of alternation in the four groups during the Y maze test (t_(14)_=1.456, *P*=0.1675) **E** The average velocity of mice during the Barnes maze test. (Interaction: F_(1, 28)_=0.03813, *P*=0.8466, Test day: F_(1, 28)_=0.02227, *P*=0.8824, Group: F_(1, 28)_=1.070, *P*=0.3098) **F** The total number of arm entry times in the four groups during the Y maze test (t_(14)_=0.2251, *P*=0.8252) **G** Experimental schematic of 808 nm NIR laser irradiation and behavior tests for naïve mice (tested on the third day after training) **H,I** Identify latency of the training period (**H**), identify latency of the test (**I**) of mice of the Barnes maze test (Interaction: F_(1, 28)_=0.2138, *P*=0.6474, Test day: F_(1, 28)_=11.70, *P*=0.0019, Group: F_(1, 28)_=0.8128, *P*=0.3750) **J** The proportion of alternation in the four groups during the Y maze test (t_(14)_=1.277, *P*=0.2224) **I** The average velocity of mice during the Barnes maze test. (Interaction: F_(1, 28)_=0.1839, *P*=0.6713, Test day: F_(1, 28)_=2.813, *P*=0.1047, Group: F_(1, 28)_=0.9832, *P*=0.3299) **J** The total number of arm entry times in the four groups during the Y maze test (t_(14)_=0.2780, *P*=0.7851) Data in **B-F** and **H-L** are mean±s.e.m., n = 8 per group. Results were analyzed by two-way repeated measures ANOVA with Bonferroni’s multiple comparisons test (**B,C,E,H,I,K**) and two tailed paired Student’s t-test (**D,F,J,L**). Statistically significant differences are indicated by asterisks: **p*<0.05.


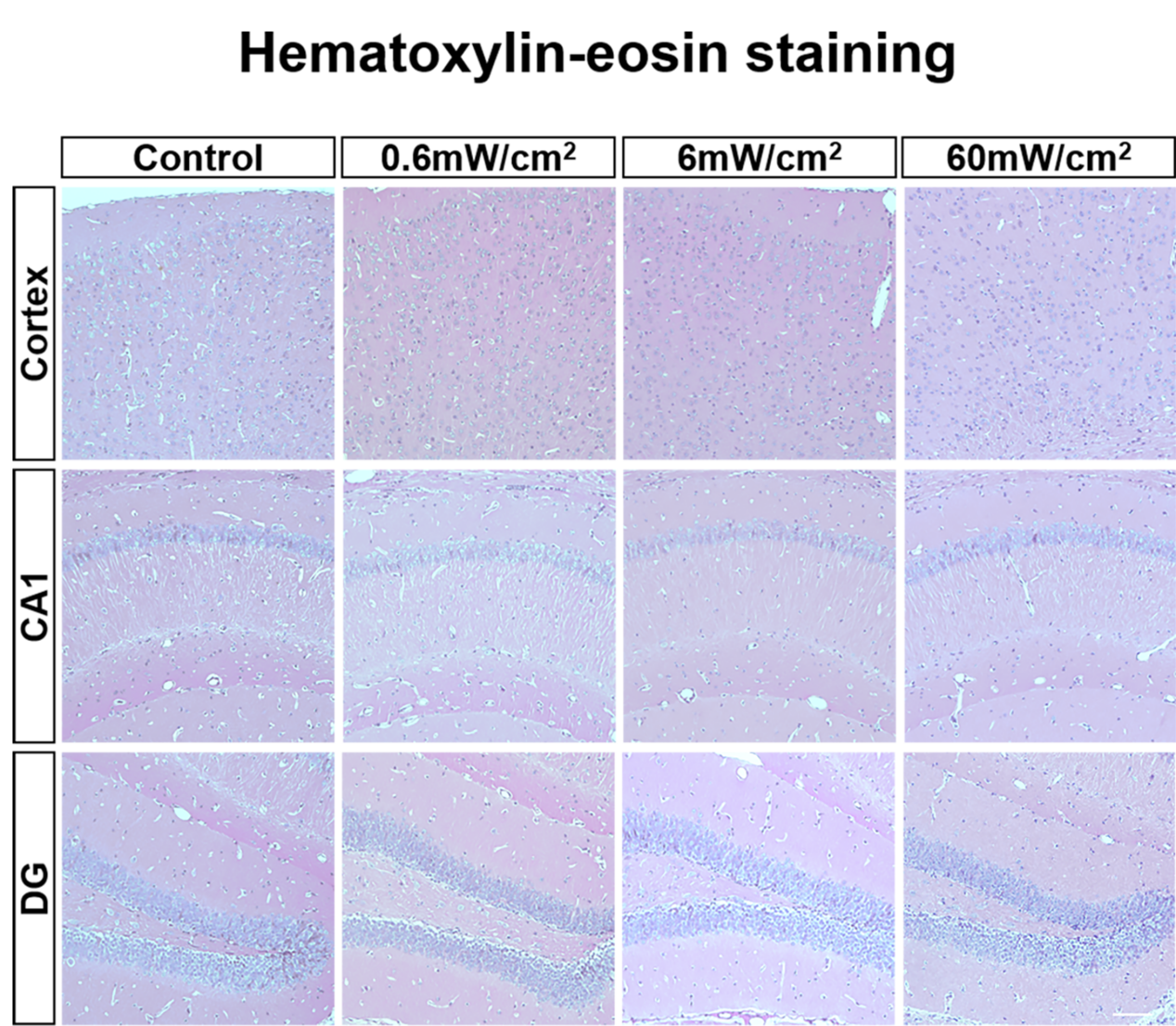


**Fig. S3. Representative histological images of cortex and hippocampus after laser irradiation.** Scale bar=100 μm.

**
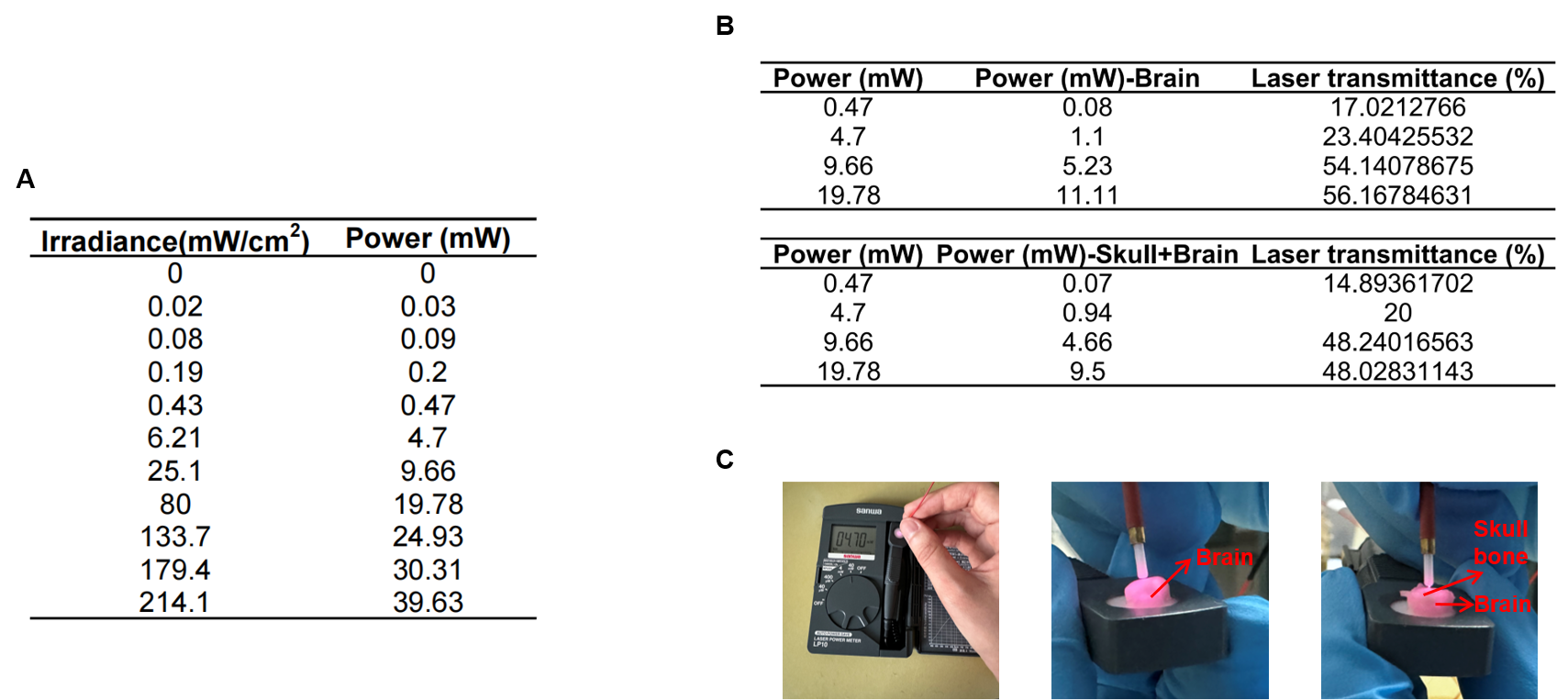
**

**Fig. S4.** **NIR instrument energy index and laser transmittance test. A** Irradiance and power comparison table for the NIR laser used in the experiment. **B** Penetrating power and laser transmittance of NIR laser with different powers to brain tissue and brain tissue composite skull. **C** Actual photographs of laser irradiation experiments.


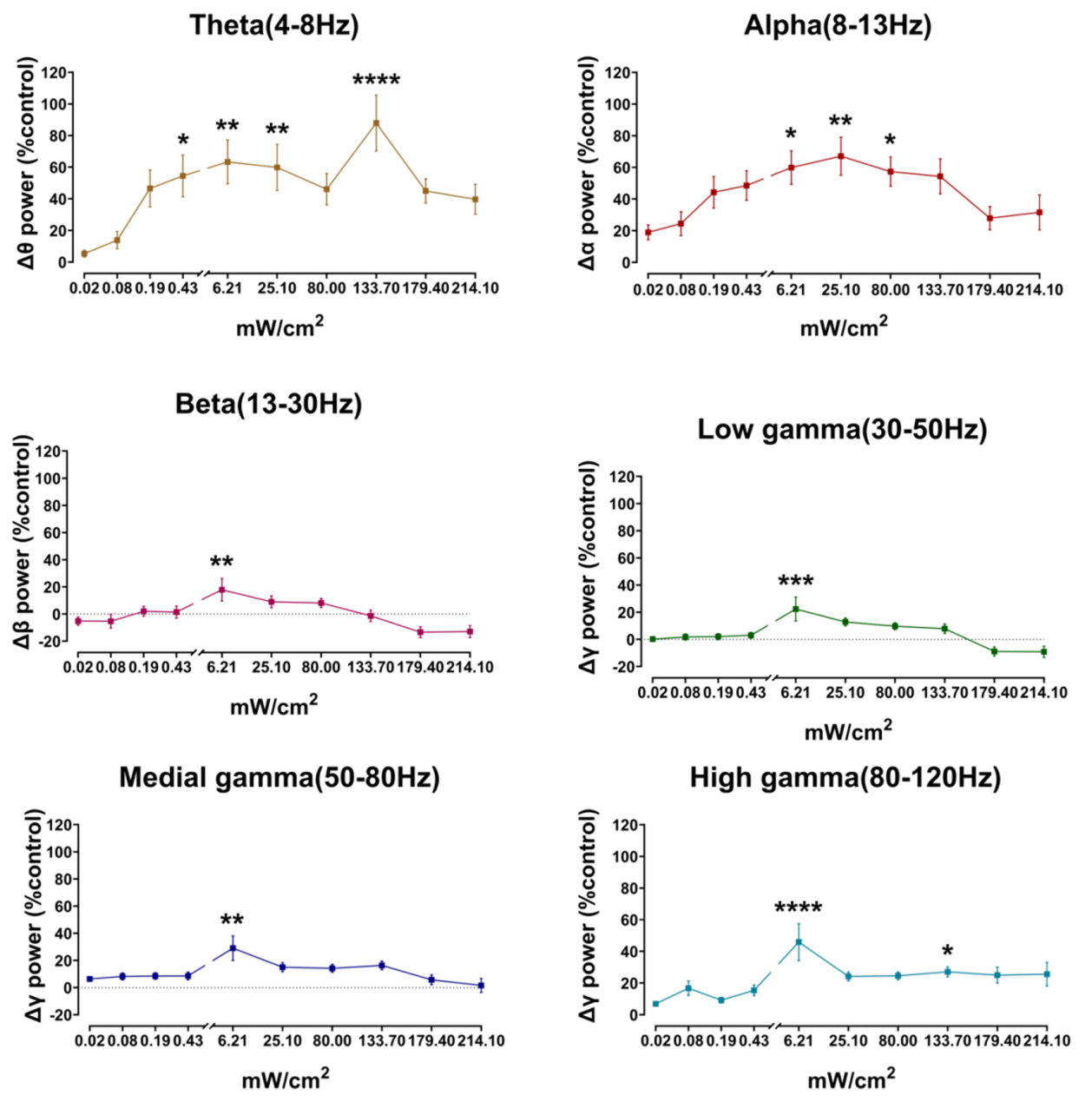


**Fig.S5.** **Analysis of local field potentials in the DG.**

For calculating the change of energy of bands from 0 to 120 s under different irradiance was derived by calculating (Energy_Irradiance_ - Energy_Baseline_)/Energy_Baseline_ for each mouse. The Energy_Baseline_ value was selected as the energy of bands when the mouse was initially exposed to no laser. The energy of bands_Irradiance_ value was the energy of bands when the same mouse was exposed to NIR laser. Data are mean±s.e.m., n = 8 per group.


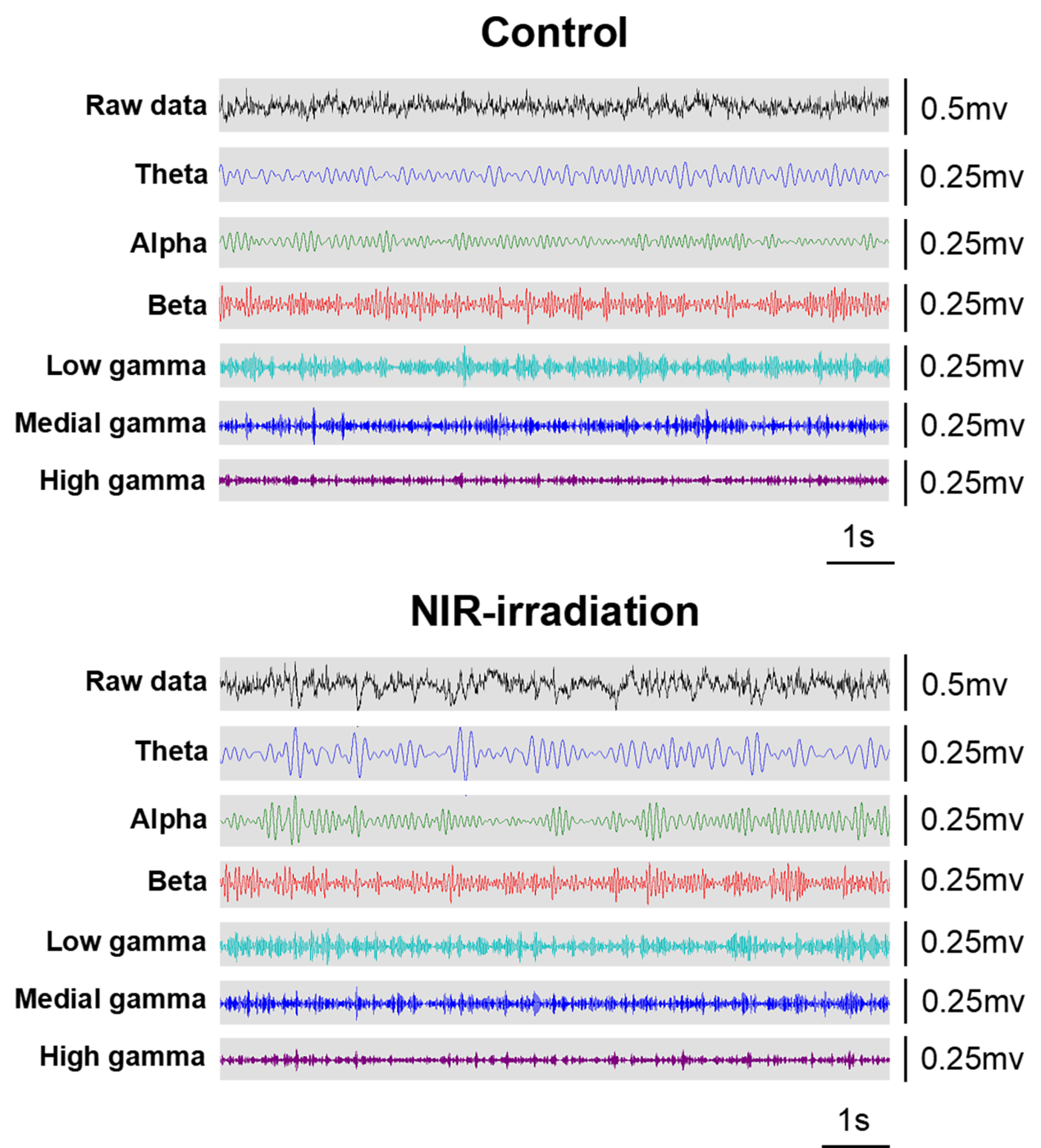


**Fig. S6. Representative traces of high-pass-filtered LFPs of control and NIR-irradiation groups.**


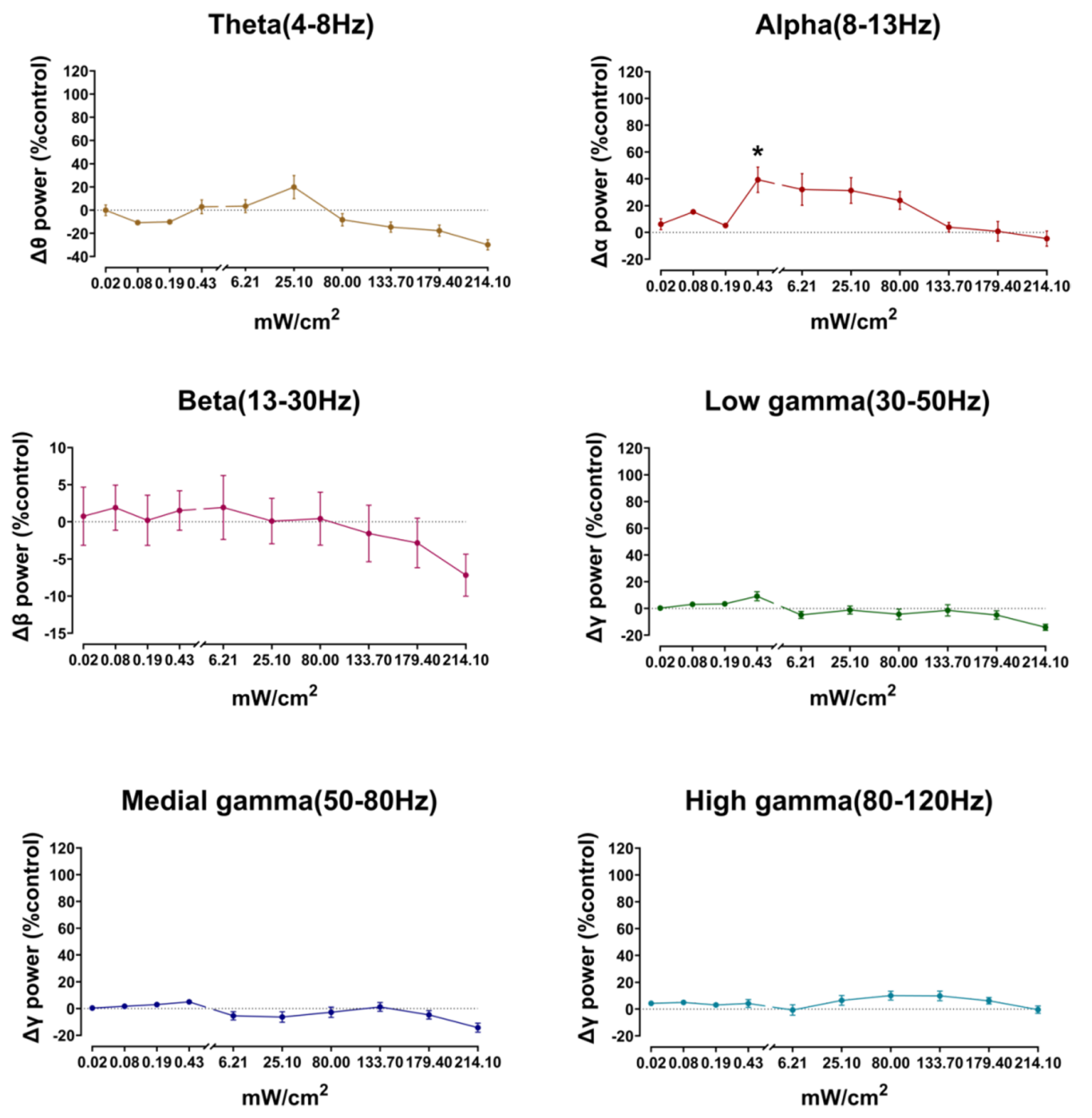


**Fig. S7.** **Analysis of local field potentials in the CA1.**

For calculating the change of energy of bands from 0 to 120 s under different irradiance was derived by calculating (Energy_Irradiance_ - Energy_Baseline_)/Energy_Baseline_ for each mouse. The Energy_Baseline_ value was selected as the energy of bands when the mouse was initially exposed to no laser. The energy of bands_Irradiance_ value was the energy of bands when the same mouse was exposed to NIR laser. Data are mean±s.e.m., n = 8 per group.


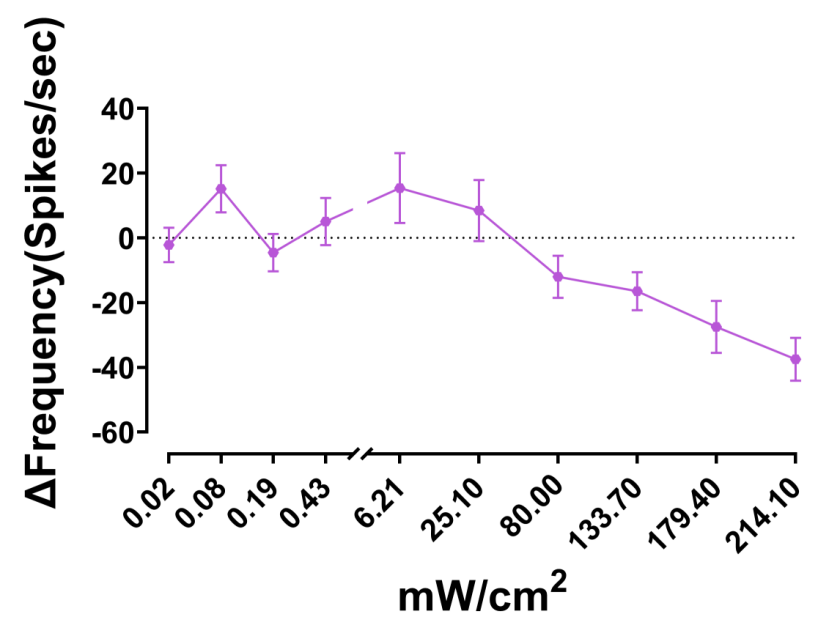


**Fig. S8. Calculation of the change of spikes in DG.** For calculating the change of spikes from 0 to 120 s under different irradiance was derived by calculating (Frequency_Irradiance_ - Frequency_Baseline_)/Frequency_Baseline_ for each mouse. The Frequency_Baseline_ value was selected as the rate of spikes change when the mouse was initially exposed to no laser. The Frequency_Irradiance_ value was the rate of change in spikes when the same mouse was exposed to NIR laser. Data are mean±s.e.m., n = 8 per group.


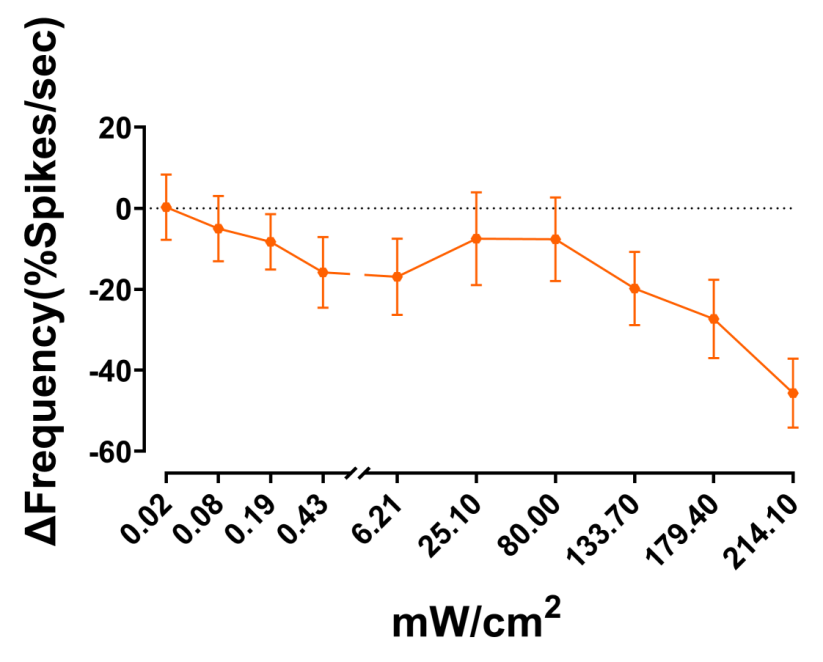


**Fig. S9. Calculation of the change of spikes in CA1.** The calculation method was shown in **Fig.S8** above. Data are mean±s.e.m., n = 8 per group.


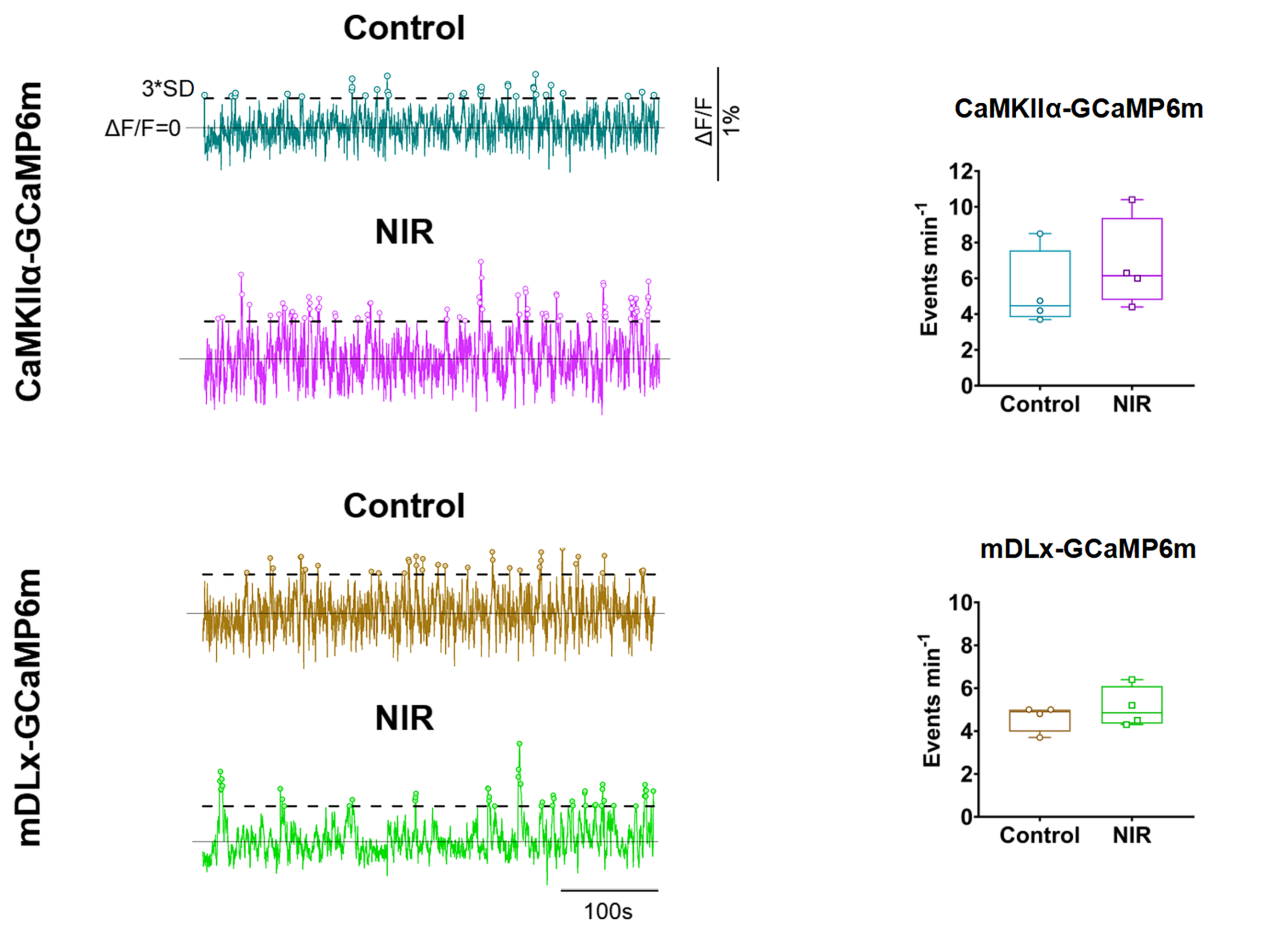


**Fig. S10. Population Ca^2+^ activity in DG neurons during Y-maze (8 min). Left:** Representative traces; **Right:** Events of population Ca^2+^ activity in DG. Data are mean±s.e.m., n = 4 per group. Results were analyzed by two tailed paired Student’s t-test (**Right**).


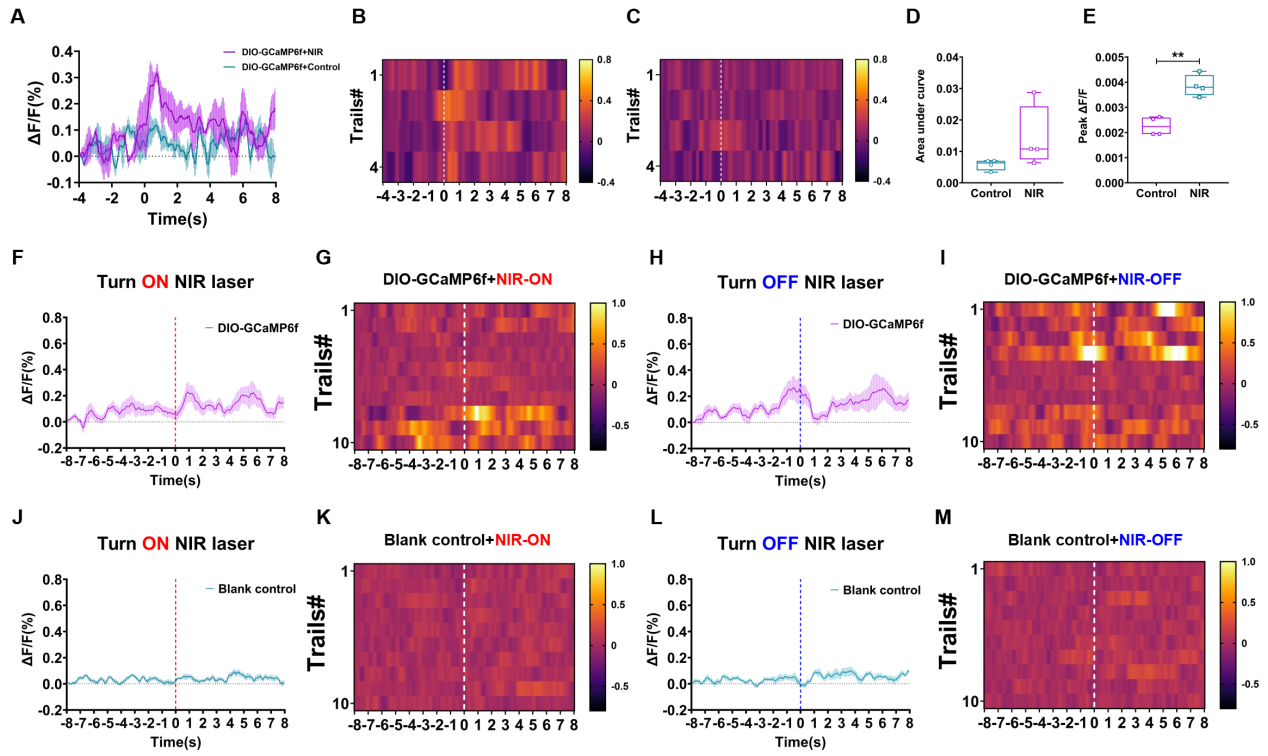


**Fig. S11. The activation effect of 808 nm NIR laser on excitatory neurons in VGluT2-ires-cre mice excluding the confounding factors. A-C** Average fluorescent Ca^2+^ signals and heat-maps in DG excitatory neurons evoked by entering the target hole in VGluT2-ires-cre mice. **D-E** Quantification of fluorescent Ca^2+^ signals’ AUC (**D**) and peak (**E**) in DG excitatory neurons in VGluT2-ires-cre mice. **F-I** Average fluorescent Ca^2+^ signals and heat-maps in DG excitatory neurons evoked by turning on/off NIR laser in VGluT2-ires-cre mice. **J-M** Average fluorescent Ca^2+^ signals and heat-maps in DG excitatory neurons evoked by turning on/off NIR laser in virus-free normal mice. Data in (**A,D,E,F,H,J,L**) are mean±s.e.m., n = 4 per group for (**A-E)**, n = 8 per group for (**F-M)**. Results were analyzed by two tailed paired Student’s t-test (**D,E**). Statistically significant differences between control and NIR laser groups are indicated by asterisks: ***p*<0.01.


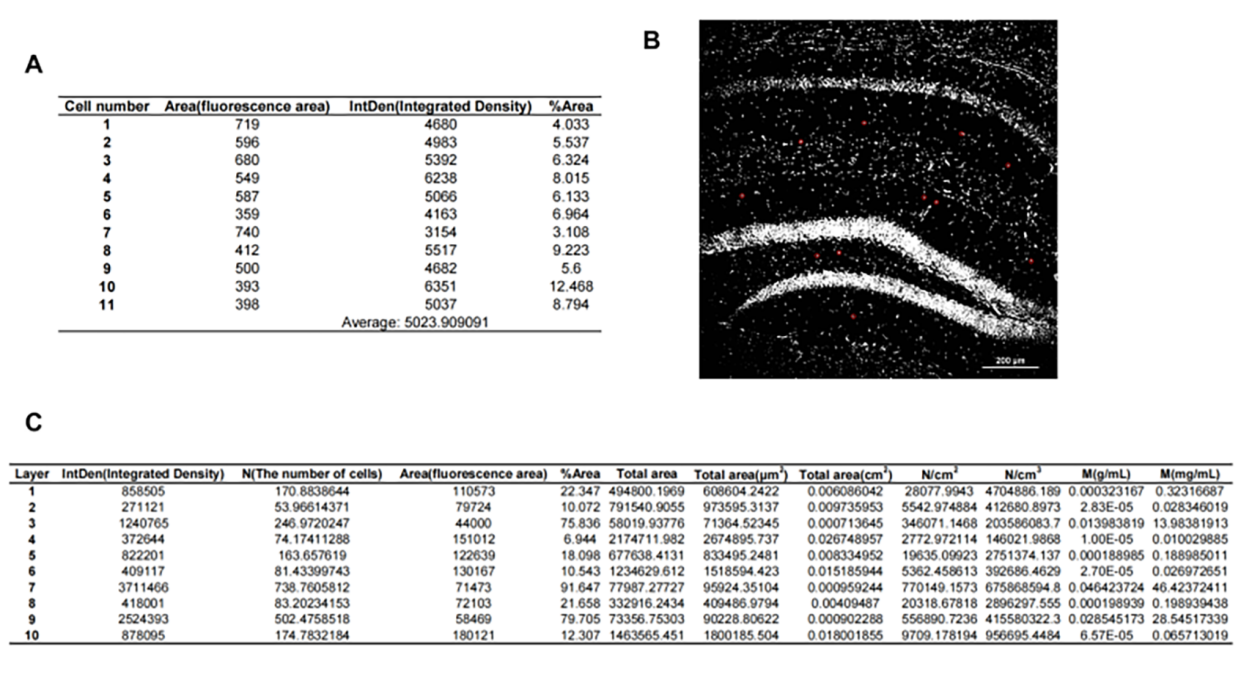


**Fig. S12 Calculation of PS microspheres concentration according to the hippocampus slices.**

DAPI (4',6-diamidino-2-phenylindole) is a fluorescent dye that binds strongly to DNA and is commonly used in fluorescence microscopy to observe the nucleus. Therefore, DAPI can reflect the number and distribution of cells laterally. In our experiment, after staining hippocampal sections with DAPI, high-resolution fluorescence photos were obtained by confocal microscopy, and statistical analysis was performed by software, and the corresponding particle concentration is converted. The details are as the following five steps:

Step 1: Brain slices were stained with DAPI and fluorescent images of the mouse hippocampal brain region were obtained using confocal laser scanning microscopy. We first selected 10 nuclei of uniform size in the plane image of mouse hippocampal brain region, and image J calculated their intden (total fluorescence intensity), so as to obtain the average fluorescence intensity of a single cell is 5023.909091.(**Fig. A, B**)

Step 2: We further manually divided the brain plane to be simulated into 1-10 layers based on the DAPI density and calculated the intden of each layer. Divide the intden of each layer by the average fluorescence intensity of individual cells obtained in step 1 to find the number of cells in each layer N.

Step 3: Image J can display the fluorescence Area (Area) and the percentage of fluorescence area (% Area) of each layer. The total area of each layer is calculated according to "Total area (S) = fluorescent area/percentage of fluorescent area". Since the area unit calculated by the data in the software is pixel, the centimeter is 1 pixel=1.24 μm=1.24×10cm^-3^ (according to the original scale of the image).

Step 4: The average proportion of a single nucleus in the total area can reflect the average number of cells per unit area.

N_S_=N/S=the number of cells per layer N/ the total area of each layer S(cm^2^), and then the average number of nuclei per unit volume is:

N_V_=$N/V$=$\left( \sqrt{N/S} \right)^{3}$

Step 5: According to the above calculation of the average number of nuclei per unit volume of each layer to calculate the corresponding microsphere concentration, according to the number of unit volumes (N_V_) to calculate the mass of polystyrene microsphere per unit volume M_V_ (diameter of 5 μm polystyrene microsphere, the unit is g/cm^3^),

M_V_=$\frac{4}{3}\pi\rho r^{3}$×N_V_,

where, $\rho$=1.05g/cm^3^, r= 2.5×10^-4^cm, the required microsphere concentration is obtained. (**Fig. C**)

(**A**) Statistical table of cell fluorescence intensity from eleven single cell, (**B**) DAPI stained high-resolution fluorescence photo obtained by confocal microscopy and (**C**) statistical table of PS microsphere concentration from 1 to 10 layers.

**
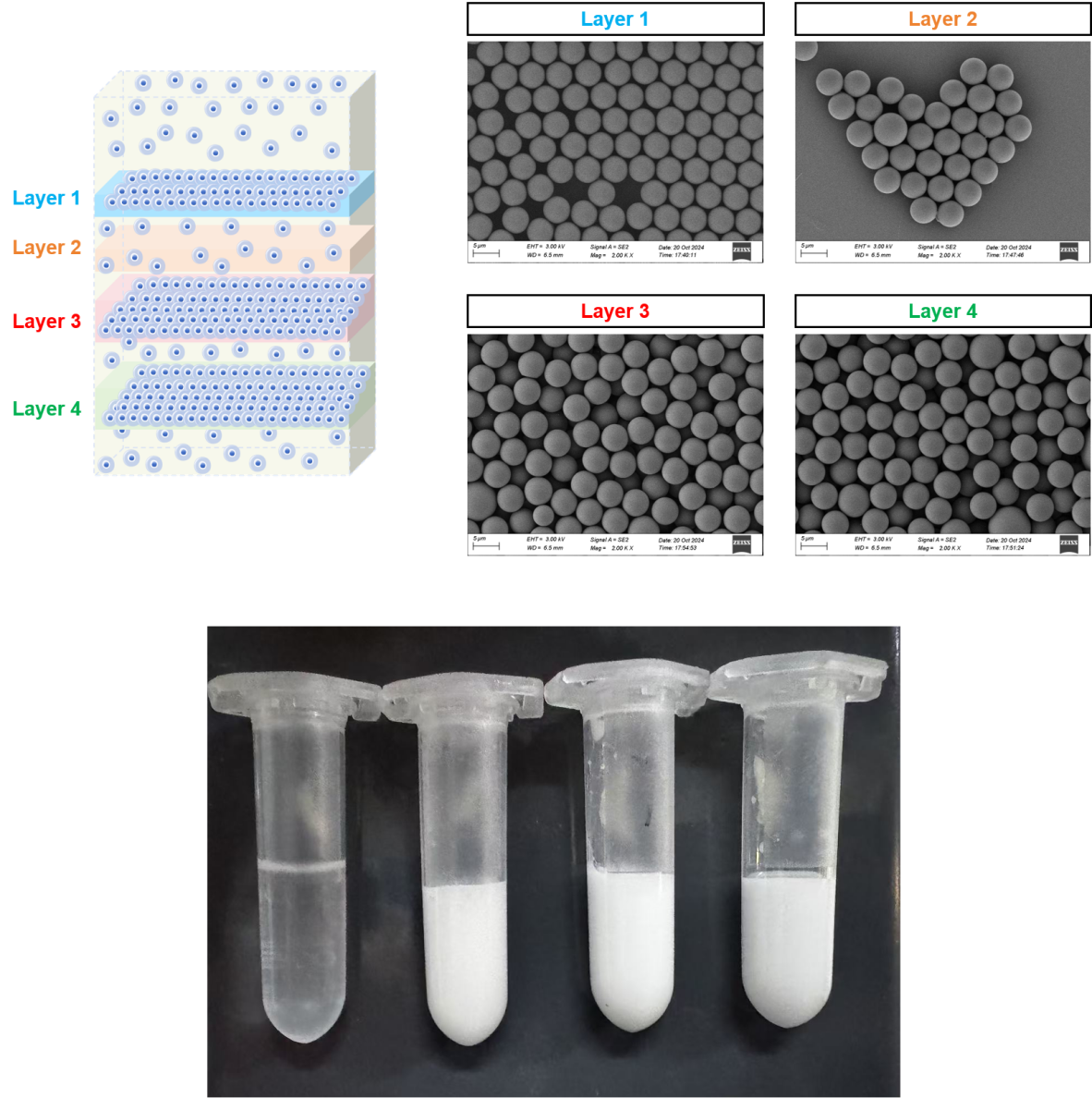
**

**Fig. S13. SEM (scanning electron microscope) images and real shots of the prepared PS microsphere solution.**


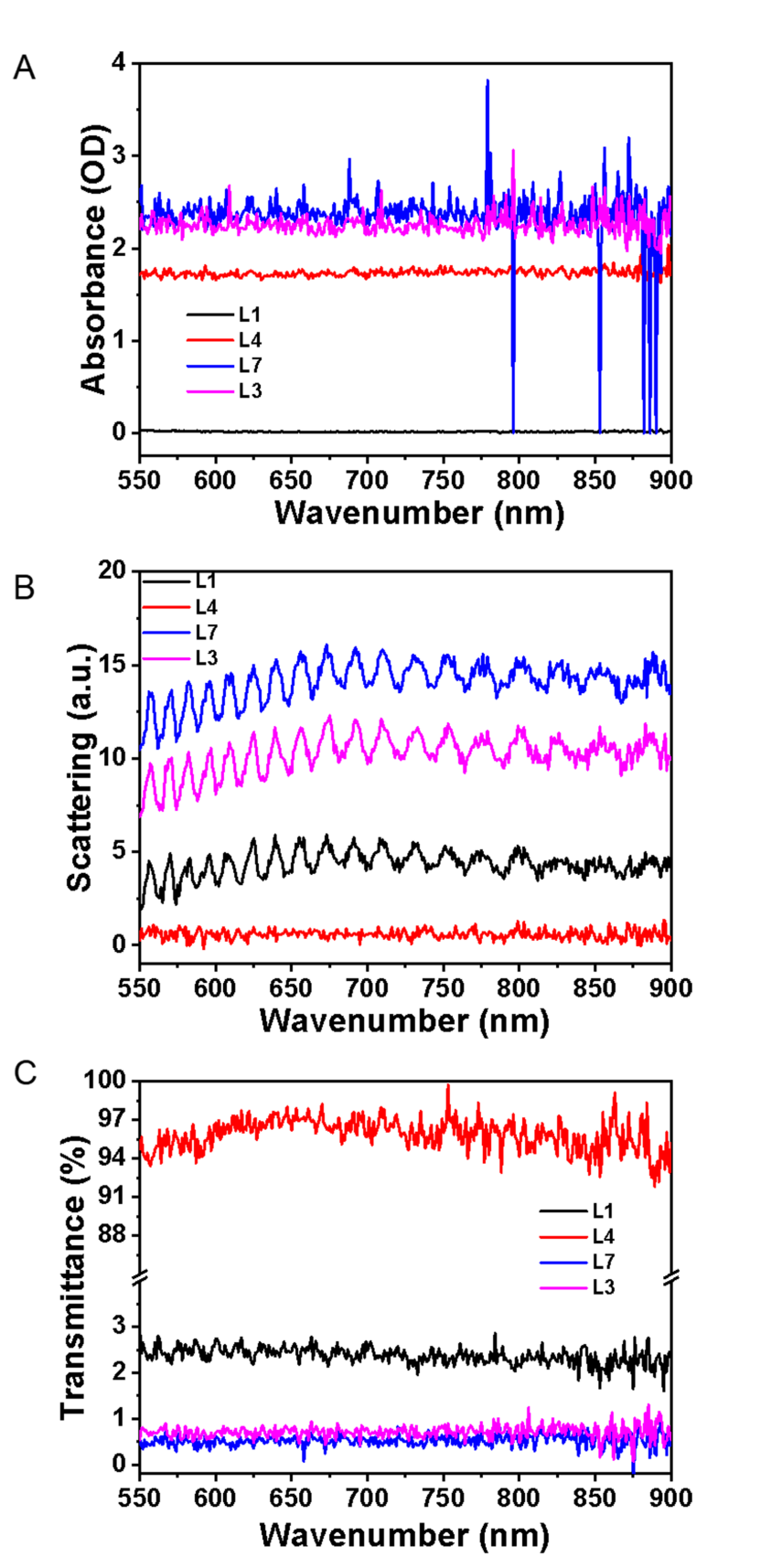


**Fig. S14. The optical characterization of PS microspheres ranging from 550 nm to 900 nm of visible-near-infrared waveband.** The absorbance (**A**), scattering (**B**) and transmittance (**C**) spectrum of PS microspheres suspension with four typical concentrations according to the cell density of hippocampus from 550 nm to 900 nm.


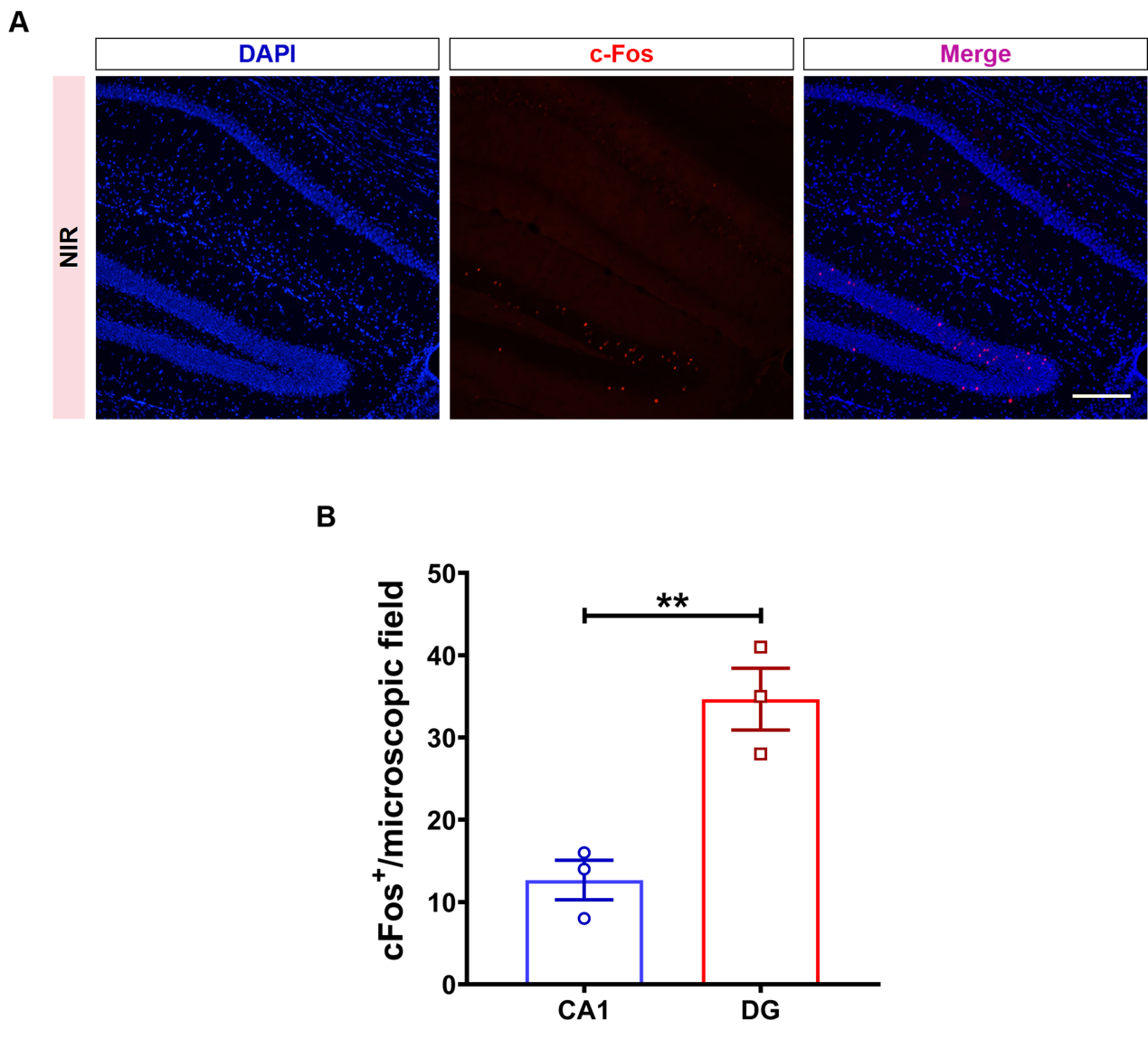


**Fig. S15. Expression of c-Fos in the hippocampus of mice after 1064 nm NIR laser irradiation. A** Representative immunofluorescence images of c-Fos (red) staining in CA1 and DG of mice (scale bar, 200 μm). **B** Quantitative data of the number of c-Fos positive cell in the CA1 and DG of mice. Data in **B** is mean±s.e.m., n = 3 per group. Results were analyzed by two tailed paired Student’s t-test. Statistically significant difference between CA1 and DG is indicated by asterisks: ***p*<0.01


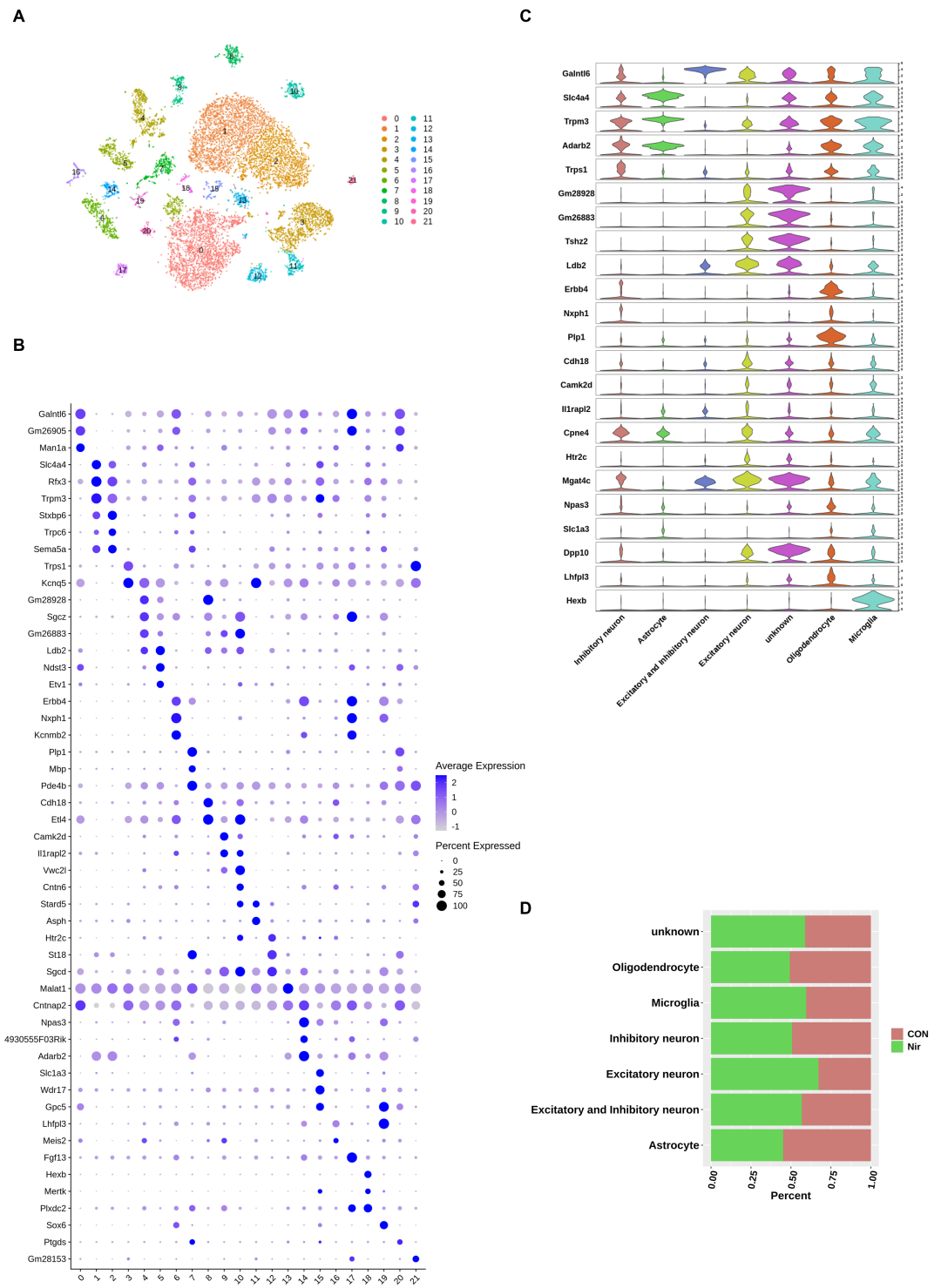


**Fig. S16. Single-cell RNA sequencing results of cells in DG (1). A,B** Visualization of t-SNE for all clusters (samples are presented separately, 16216 cells) Dots, individual cells; colors, neuron clusters. Group: NIR and CON group. **C** Top 3 genes were selected for heat mapping in all clusters. The graph shows marker genes in the horizontal line and cells in the vertical line, and the top color bars represent different clusters. **D** Violin plots of significantly characterized genes for each cluster. **E** Statistical plots of percentages of different samples from each cluster.


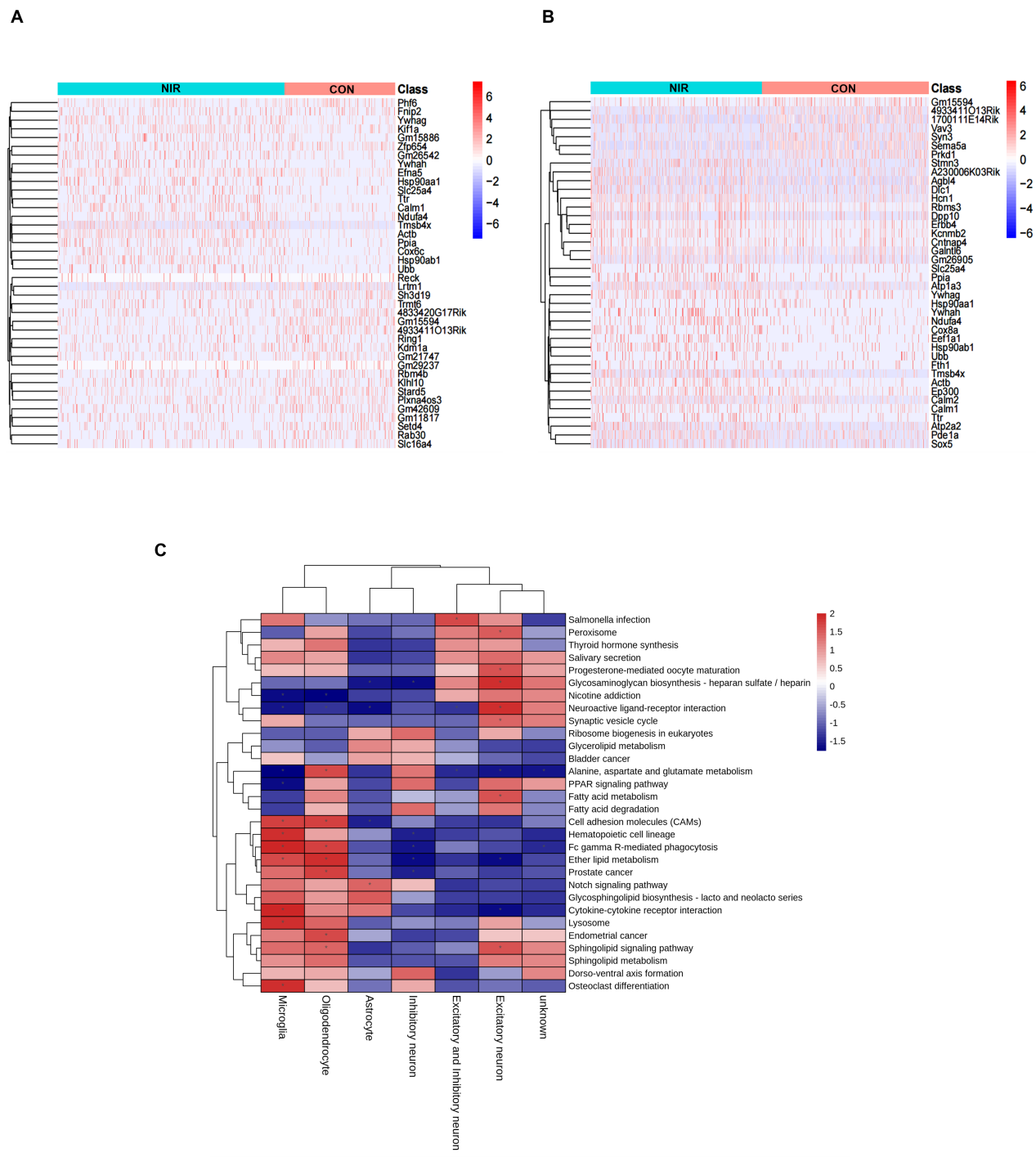


**Fig. S17. Single-cell RNA sequencing results of cells in DG (2). A** Heatmap analysis of differentially expressed genes between NIR and CON groups in the cluster of excitatory neurons. **B** Heatmap analysis of differentially expressed genes between NIR and CON groups in the cluster of inhibitory neurons. **C** The GSEA analysis heatmap is shown as follows, showing only the three gene sets (pathways) with the largest NES values in each cluster.


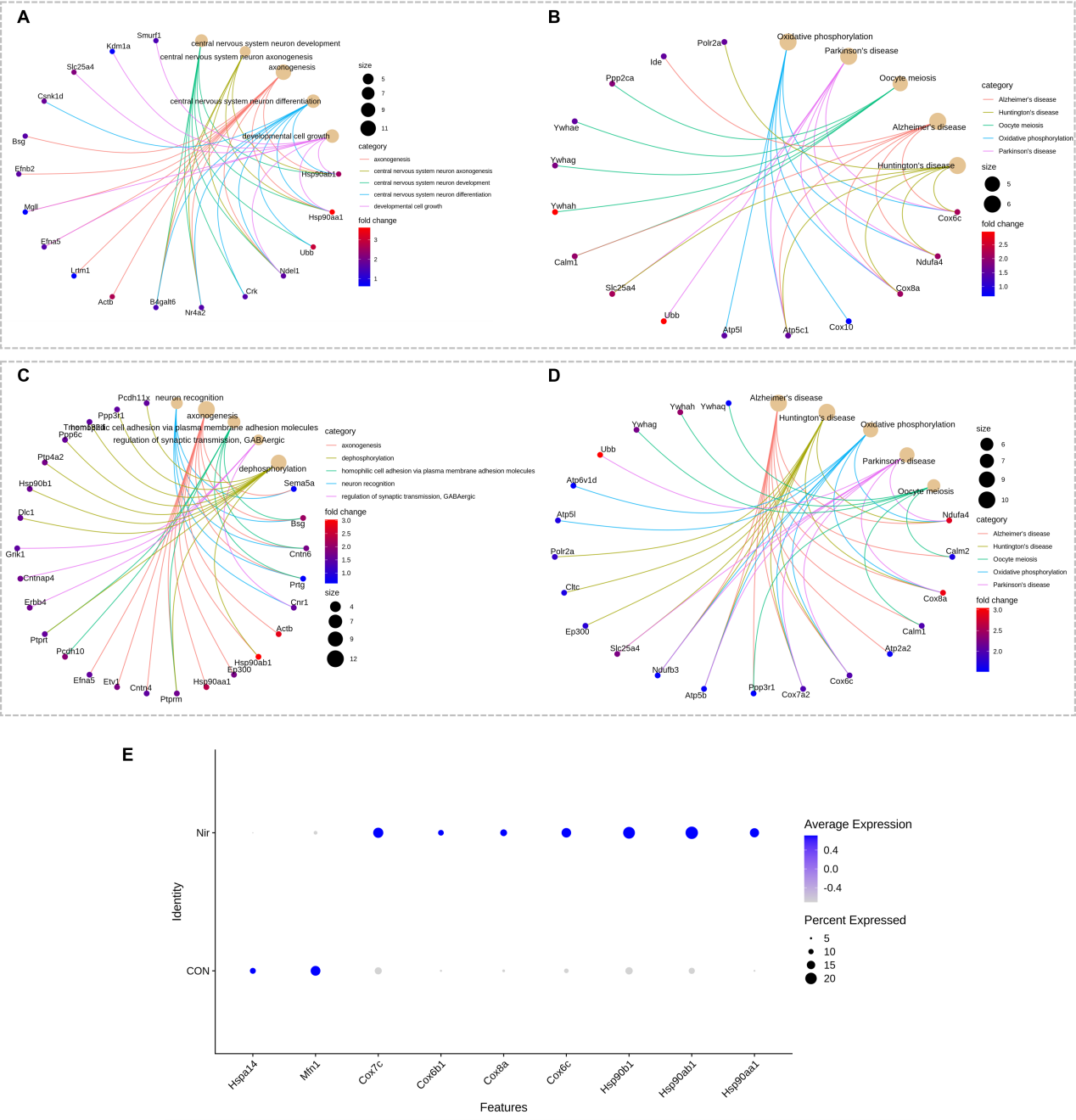


**Fig. S18. Single-cell RNA sequencing results of cells in DG (3).**  **A** Network diagram of GO enrichment of marker genes of excitatory neuron. **B** Network diagram of KEGG enrichment of marker genes of excitatory neuron. **C** Network diagram of GO enrichment of marker genes of inhibitory neuron. **D** Network diagram of KEGG enrichment of marker genes of inhibitory neuron. **E** Significantly differentially expressed genes related to cytochrome c oxidase and mitochondrial metabolism between NIR and CON groups.


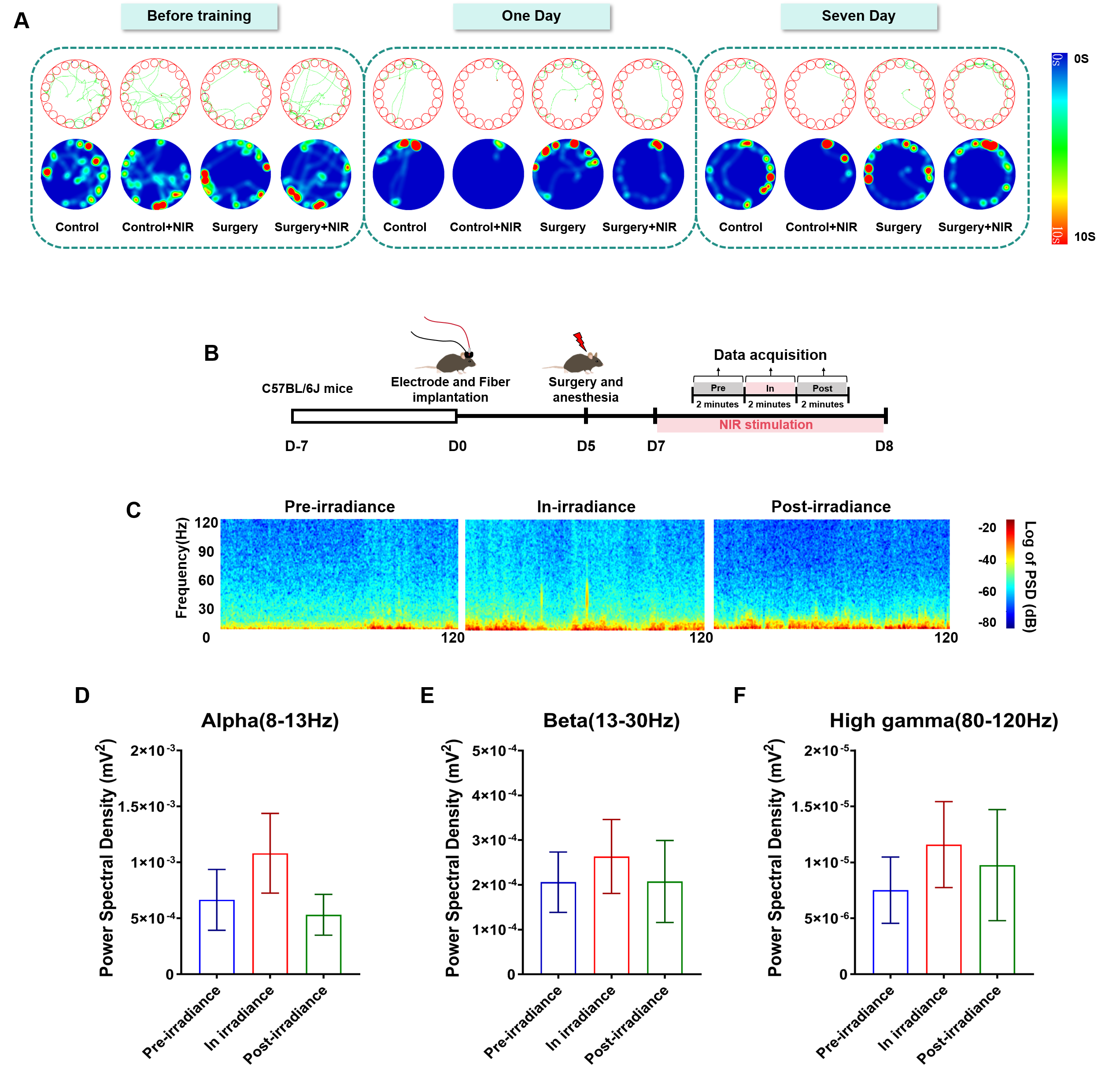


**Fig. S19. 808 nm NIR laser rescued cognitive dysfunction and improved the energy of multiple frequency and theta-gamma coupling of DG of POCD model mice. A** Typical movement trajectory of mice during the Barnes maze test. **B** Experimental timeline and schematic diagram showing multi-channel recordings in the DG of head-fixed conscious mice. **C** Representative heat-maps of the power spectrogram pre-, in-, and post- NIR irradiance recording from the DG. **D-F** The power of oscillations pre-, in-, and post- NIR irradiance recording from the DG. D to F is alpha, beta and high-frequency gamma oscillations in order. Data in **D-F** are mean±s.e.m., n = 8 per group. Results were analyzed by Kruskal-Wallis ANOVA with a *post hoc* Dunn's test **(D-F)**.


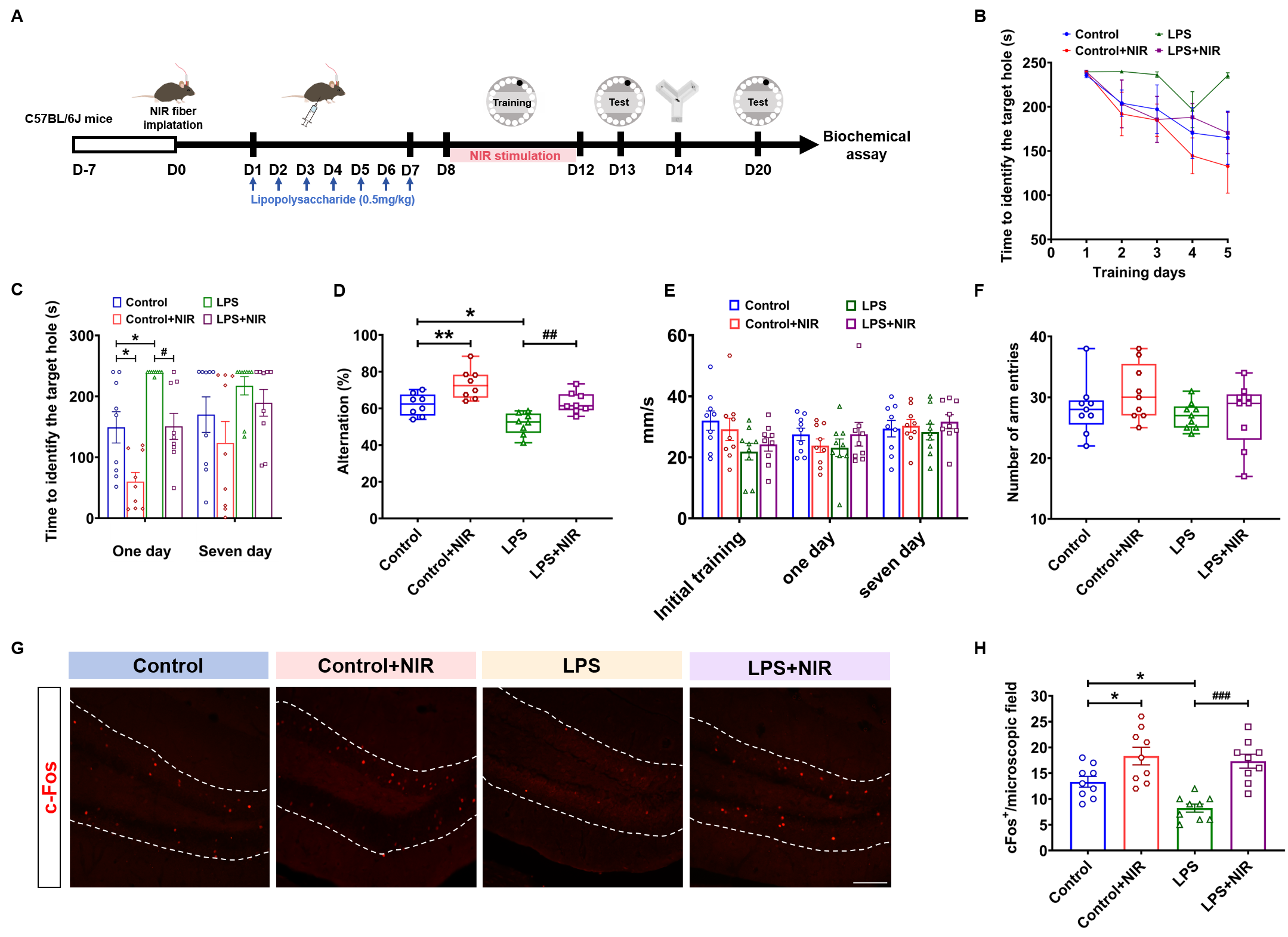


**Fig. S20. 808 nm NIR laser rescued cognitive dysfunction of LPS-induced cognitive dysfunction model mice. A** Experimental schematic of 808 nm NIR laser irradiation and behavior tests (LPS-induced cognitive dysfunction model). **B,C** The identify latency of the training period and identify latency of the test of mice during the Barnes maze test in LPS-induced cognitive dysfunction model mice. **D** The proportion of alternation in the four groups during the Y maze test. (F_(3, 28)_=14.35, *P*<0.0001) **E** The average velocity of mice during the Barnes maze. (Interaction: F_(6, 64)_=0.9829, *P*=0.4443, Test day: F_(1.900, 60.79)_=2.720, *P*=0.0766, Treatment: F_(3, 32)_=1.774, *P*=0.1719). **F** The total number of arm entry times in the four groups during the Y maze test. (F_(3, 32)_=1.400, *P*=0.2608) **G** Representative immunofluorescence images of c-Fos (red) staining in the DG of mice. (scale bar, 100 μm) **H** Quantitative data of the number of c-Fos positive cell in the DG of mice (F_(3, 32)_=13.17, *P*<0.0001). Data in **B,C-F,H** are mean±s.e.m., n = 9 per group. Data were analyzed by two-way repeated measures ANOVA with Bonferroni’s multiple comparisons test (**B,C,E**) and one-way repeated measures ANOVA with Bonferroni’s multiple comparisons test (**D,F,H**). Statistically significant differences between control and other groups are indicated by asterisks: **p*<0.05, ***p*<0.01. Statistically significant differences between LPS and other groups are indicated by the pound sign: ^#^*p*<0.05, ^##^*p*<0.01, ^###^*p*<0.001.

**
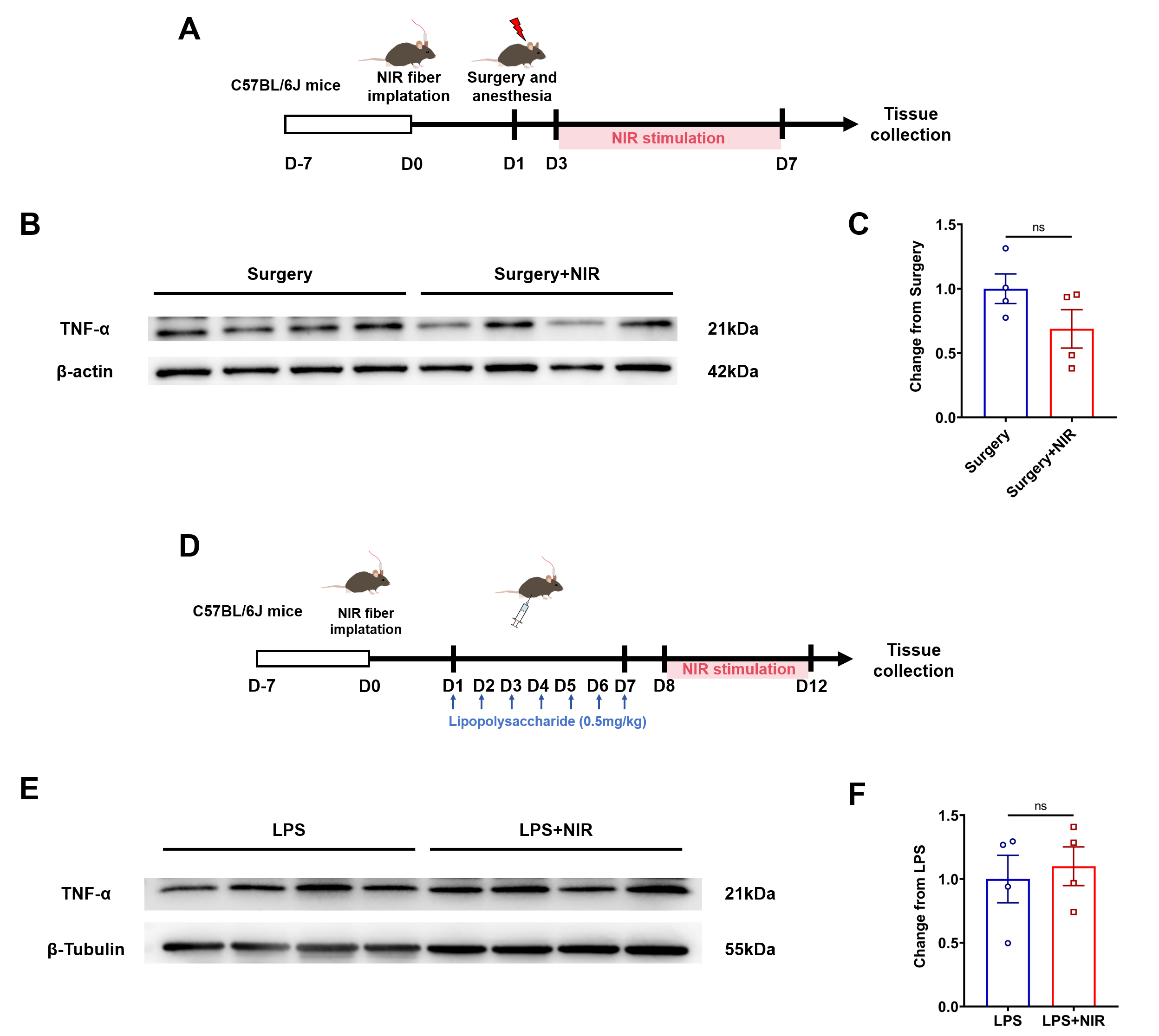
**

**Fig. S21. Changes in TNF-α in the DG of cognitive dysfunction model mice.** **A** Experimental schematic of 808 nm NIR laser irradiation and tissue collection (POCD model). **B** Representative western blot and **C** the corresponding bar graphs with dots showing TNF-α expression levels in the DG of POCD model mice irradiated with or without laser irradiation (t(6)=1.655, P=0.1490). **D** Experimental schematic of 808 nm NIR laser irradiation and tissue collection (LPS-induced cognitive dysfunction model). **E** Representative western blot and **F** the corresponding bar graphs with dots showing TNF-α expression levels in the DG of LPS-induced cognitive dysfunction model mice irradiated with or without laser irradiation (t(6)=0.4182, P=0.6904).
